# Hydraulic performance of 14-G hemodialysis catheters versus an 8-Fr arterial cannula in a mock 3-kg small-dog cardiopulmonary bypass circuit

**DOI:** 10.1371/journal.pone.0361473

**Published:** 2026-10-26

**Authors:** Keizo Wauchi, Chisato Ikubo, Hirohito Imada, Masahiro Miyata, Kenji Tani

**Affiliations:** 1 Department of Medical Technology, Faculty of Fukuoka Medical Technology, Teikyo University, Omuta, Fukuoka, Japan; 2 Laboratory of Veterinary Surgery, Joint Faculty of Veterinary Medicine, Yamaguchi University, Yamaguchi, Yamaguchi, Japan; 3 Graduate Degree Program of Comprehensive Applied Data Science, Teikyo University, Hachioji, Tokyo, Japan; 4 Biodesign Division, Department of Academia-Government-Industry Collaboration, Hiroshima University, Hiroshima, Japan; NED University of Engineering and Technology, PAKISTAN

## Abstract

Arterial cannulation for cardiopulmonary bypass in small dogs is challenging because small vessel diameters may preclude use of appropriately sized pediatric arterial cannulas. Although off-label use of 14-gauge hemodialysis catheters has been reported, their pressure–flow characteristics under small-dog cardiopulmonary bypass conditions remain unclear. This in vitro study evaluated one 8-French pediatric arterial cannula and six 14-gauge hemodialysis catheter specimens—separate side-hole and sealed specimens at effective lengths of 25, 32, and 38 mm—in a closed-loop mock circuit for a 3-kg small dog. A 34% (w/w) glycerol–water solution (viscosity 2.8 cP at 28°C) served as an approximate Newtonian blood analogue. The Stockert S5 pump flow-rate setting was increased from 50 to 350 mL/min; 50–300 mL/min was prespecified as the primary analysis range and 350 mL/min as exploratory. The reported outlet pressure was recorded 1,000 mm upstream of the test device, while inlet pressure was monitored 300 mm downstream and regulated to 60 mmHg; six technical runs per condition were conducted using the same physical device and circuit setup. Within the primary analysis range, outlet pressure was approximately 136–137 mmHg at 300 mL/min; mean differences for the 14-G specimens versus the 8-French cannula were +0.33 to +0.67 mmHg, with 95% confidence intervals from −1.33 to +2.15 mmHg. At 350 mL/min, outlet pressures were higher for all three side-hole 14-gauge specimens than for the 8-French cannula. At 300 mL/min, outlet pressure was approximately 2–3 mmHg lower for the side-hole specimens than for the sealed specimens across all catheter lengths. Together, these findings indicate small observed circuit-level differences in outlet pressure among tested devices within this standardized mock small-dog cardiopulmonary bypass circuit, but do not establish formal equivalence, device-to-device interchangeability, or clinical validity.

## Introduction

Maintaining stable arterial pressure and flow during cardiopulmonary bypass (CPB) is essential for adequate tissue perfusion and for limiting blood trauma such as hemolysis [1,2]. In practice, arterial-line pressure reflects the net hydraulic resistance of the extracorporeal circuit, which is strongly influenced by the arterial access device and downstream tubing segment [3].

With advances in veterinary cardiac surgery and perfusion management, CPB has been increasingly applied to small dogs for correction of congenital and acquired heart disease [4–6]. These developments parallel broader trends toward miniaturized and low-flow perfusion systems and low-prime circuit concepts [7–9]. In very small patients, including toy-breed dogs of approximately 3 kg, narrow arterial calibers and low target flow rates can magnify the impact of component-level pressure losses, and even modest differences in device geometry can alter local flow patterns and shear-related stresses in the arterial line [3,10].

However, appropriately scaled pediatric arterial cannulas are not always available for small-dog CPB [4,5]. Although the 6-Fr arterial cannula (outer diameter, 2.0 mm) is geometrically closer to the 14-G catheter (outer diameter, 2.1 mm), the 6-Fr cannula could not be procured as a new device for standardized experimental testing in Japan [11]. In contrast, the 8-Fr pediatric arterial cannula has been used as an arterial access device in very small dogs and therefore served as a clinically relevant benchmark comparator in the present study [11]. Off-label use of 14-gauge (G) hemodialysis catheters as alternative arterial access devices in low-flow extracorporeal circuits has been reported [11]. However, quantitative characterization of pressure–flow behavior and side-hole effects under controlled CPB-relevant conditions remains limited, despite related computational analyses such as computational fluid dynamic simulations of dialysis catheters and cannula–vessel configurations [12,13].

Moreover, idealized tube-flow calculations do not fully capture device performance in practice. Real devices incorporate local geometric features, such as tapering tips and side holes, which introduce localized losses and flow redistribution; accordingly, outlet pressure within a standardized circuit segment cannot be inferred only from idealized calculations [3].

Therefore, this in vitro study aimed to quantify outlet pressure characteristics of an 8-French (Fr) pediatric arterial cannula and 14-G hemodialysis catheters within a standardized measured circuit segment of a closed-loop mock CPB circuit scaled to reproduce hemodynamic conditions expected for a 3-kg small dog. Specifically, we evaluated the combined hydraulic behavior of each device and the tubing between the two pressure-measurement sites under low-flow conditions representative of small-dog CPB, while systematically varying the pump flow-rate setting, effective length, and side-hole configuration. The overarching goal was to provide engineering reference data to support device selection and circuit design in small-dog CPB and, more broadly, in miniaturized low-flow perfusion systems. In this study, we hypothesized that, within the prespecified primary analysis range of the present mock small-dog CPB circuit, the observed outlet-pressure differences between the three side-hole 14-G specimens and the 8-Fr pediatric arterial cannula would be small in magnitude.

## Materials and methods

### Mock small-dog cardiopulmonary bypass circuit

A closed-loop mock CPB circuit was constructed and scaled to reproduce the hemodynamic conditions of a 3-kg small-dog circulation model (Fig 1). The system consisted of a CPB machine with a roller pump and temperature sensor (Stockert S5; LivaNova Japan KK, Tokyo, Japan), two pressure transducers (TruWave; Edwards Lifesciences, Tokyo, Japan), two physiologic monitors (BSM-2300; Nihon Kohden Corporation, Tokyo, Japan), and a temperature control unit (HHC-51; Senko Medical Instrument Mfg. Co., Ltd., Tokyo, Japan). All components were connected using polyvinyl chloride tubing with an inner diameter of 1/4 inch and tube clamps to complete the closed loop. All tubing junctions were sealed externally with cyanoacrylate adhesive to prevent leakage; the adhesive was not applied to the intraluminal surfaces of the test devices.

**Fig 1:**
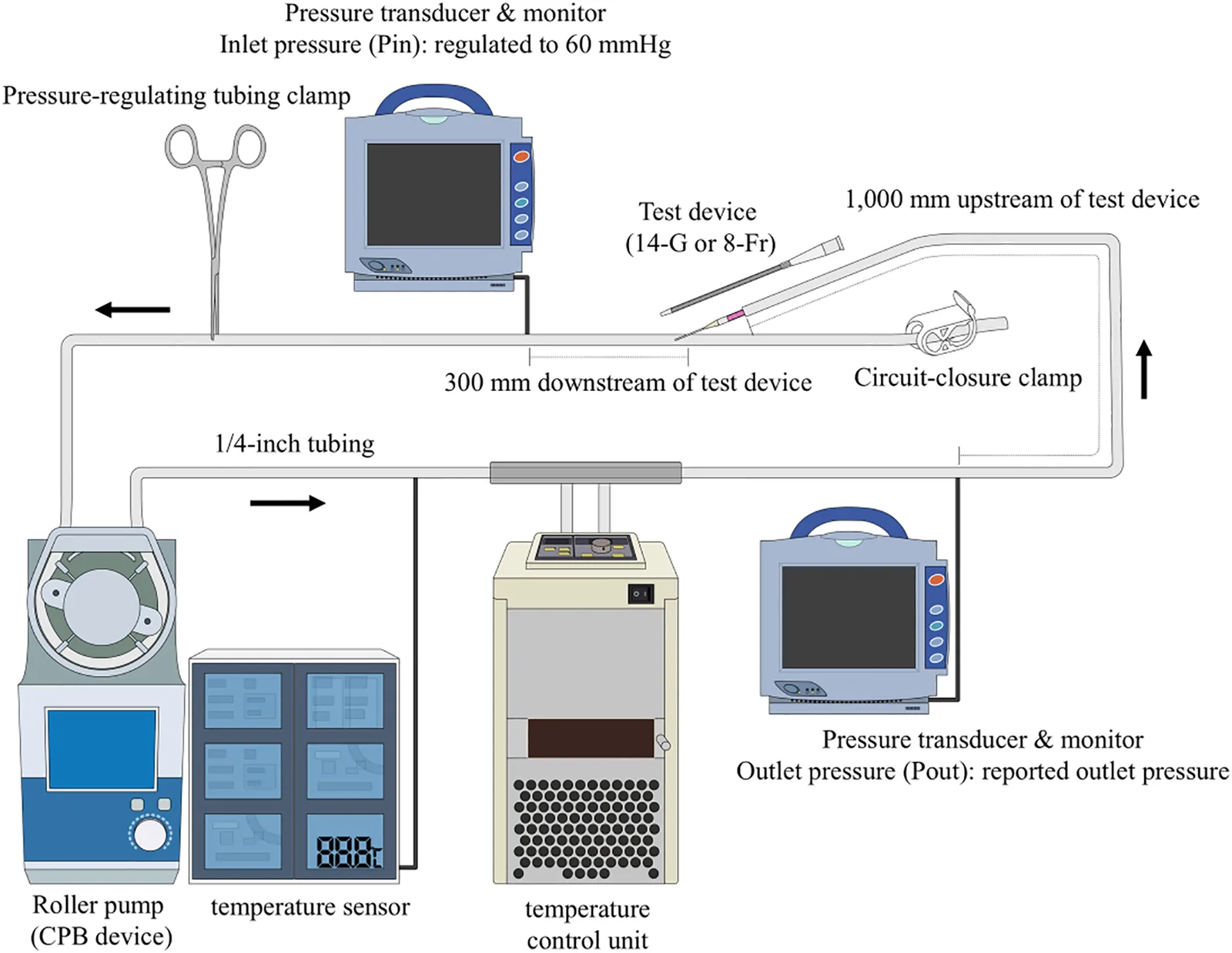
Mock small-dog cardiopulmonary bypass circuit schematic. The closed-loop circuit comprises a roller pump, polyvinyl chloride tubing with an inner diameter of 1/4 inch, tube clamps, two pressure transducers and two physiologic monitors, a temperature sensor and temperature control unit, and the test arterial access device (14-G hemodialysis catheter or 8-Fr pediatric arterial cannula). Each test device was inserted into the arterial-line tubing such that approximately 10 mm of its distal portion extended into the tubing lumen. Arrows indicate the direction of perfusate flow. Inlet pressure (*P*_in_), monitored 300 mm downstream of the test device, was regulated to 60 mmHg before each recording by manual adjustment of the pressure-regulating tubing clamp. The separate circuit-closure clamp was used only to maintain the closed-loop configuration and prevent loss of perfusate. Outlet pressure (*P*_out_), recorded 1,000 mm upstream of the test device, was the pressure outcome reported in this study.

Two types of perfusion devices were evaluated: (1) an 8-Fr pediatric arterial cannula (8-Fr; one-piece design; Medtronic Japan Co., Ltd., Tokyo, Japan) and (2) 14-G hemodialysis catheters (SafeTouch Cannula GA; Nipro Corporation, Osaka, Japan) trimmed to effective lengths of 25, 32, and 38 mm (hereafter referred to as 14-G 25, 14-G 32, and 14-G 38, respectively). One physical 8-Fr arterial cannula and six physical 14-G hemodialysis catheters were used. For each effective length (25, 32, and 38 mm), one catheter was tested with the original side holes and a separate catheter was tested in the sealed condition, resulting in six 14-G test specimens and seven physical test devices in total. Each test device was inserted into the arterial-line tubing at the test-device site shown in Fig 1, such that approximately 10 mm of the distal portion of the device extended into the tubing lumen. Inlet pressure (*P*_in_) was monitored 300 mm downstream of the test device and regulated to 60 mmHg, whereas the reported outlet pressure (*P*_out_) was recorded 1,000 mm upstream of the test device. In this study, *P*_in_ and *P*_out_ denote the designated circuit pressure-monitoring sites and do not refer to the inlet and outlet of the test device. A pressure-regulating tubing clamp was located downstream of the *P*_in_ measurement site, whereas a separate circuit-closure clamp was used only to maintain the closed-loop configuration and prevent loss of perfusate. These measurement sites are labeled in Fig 1, and arrows indicate the direction of perfusate flow. This configuration was intended to represent arterial-line pressure monitoring in small-dog CPB [4–6]. Accordingly, the calculated pressure difference between the recorded *P*_out_ value and the regulated *P*_in_ target reflects the combined hydraulic behavior of the test device and the tubing between the two pressure-measurement sites rather than device-intrinsic resistance alone. The 60-mm length used for geometric comparison for the 8-Fr arterial cannula corresponded to the measured distal narrow-bore segment extending to the cannula tip and did not represent the overall device length. In contrast, for the 14-G catheters, effective length was defined as the axial distance from the catheter inlet to the distal tip/side-hole region. The selected catheter effective lengths were intended to represent device-length configurations relevant to toy-breed and small-dog applications.

The circuit was primed with a 34 wt% glycerol–water solution. The mean ± standard deviation (SD) dynamic viscosity of the working fluid—measured at 28°C using a tuning-fork vibration viscometer (SV-10; A&D Company, Tokyo, Japan)—was 2.80 ± 0.05 cP (*n* = 6). Because blood viscosity varies with hematocrit and exhibits non-Newtonian behavior [14,15], the working fluid was used only as an approximate Newtonian blood analogue for standardized hydraulic testing and was not intended to reproduce the species- and temperature-specific rheology of hypothermic, hemodiluted canine blood. These conditions minimized cellular and non-Newtonian effects, facilitating evaluation of geometric and hydraulic contributions to pressure loss. Device dimensions, including the length used for geometric comparison and inner diameter, are summarized in Table 1. Manufacturer documentation was also reviewed for device flow specifications. The documentation reviewed did not specify a maximum recommended blood-flow rate for either tested device. Medtronic provides pressure-loss curves as a function of water flow for the pediatric one-piece arterial cannulae, whereas Nipro reports an average flow of 343 mL/min for the 14-G SafeTouch Cannula GA measured at an effective length of 38 mm; this value represents an average flow specification rather than a maximum-flow limit [16,17].

**Table 1:** Geometric characteristics and manufacturer-reported flow information for the arterial cannula and 14-G hemodialysis catheters.

Device	Length used for geometric comparison, *L*	Outer diameter, *D*_o_	Inner diameter, *D*_i_	Side holes, *n*	Manufacturer-reported flow information
	[mm]	[mm]	[mm]	[n]	
**Arterial cannula (Pediatric one-piece)**					
**6-Fr (reference only)**	60	2.0	1.3	0	Reference only
**8-Fr**	60	2.7	2.0	0	Maximum not specified; pressure-loss curve provided
**Hemodialysis catheter (SafeTouch cannula GA)**					
**14-G 25**	25	2.1	1.6	4	Maximum not specified
**14-G 32**	32	2.1	1.6	4	Maximum not specified
**14-G 38**	38	2.1	1.6	4	Average flow, 343 mL/min; maximum not specified

Note: 8-Fr represents the pediatric arterial cannula, and 14-G 25, 14-G 32, and 14-G 38 represent 14-G hemodialysis catheters trimmed to effective lengths of 25, 32, and 38 mm, respectively. For the 8-Fr cannula, the 60-mm value represents the measured distal narrow-bore segment used for geometric comparison rather than the overall device length. The 6-Fr cannula is included for specification reference only and was not used for experimental testing because it could not be procured as a new device for standardized bench testing in Japan [11]. Manufacturer documentation did not specify a maximum recommended blood-flow rate for either tested device. For the pediatric one-piece arterial cannula, Medtronic provides pressure-loss curves as a function of water flow rather than a maximum recommended blood-flow rate [16]. For the 14-G SafeTouch Cannula GA, Nipro reports an average flow of 343 mL/min based on a 38-mm effective length; this value is not specified as a maximum recommended flow rate [17].

### Circuit scaling and target flow for the 3-kg small-dog model

Body surface area (BSA) was estimated from body weight using Equation 1 [18]:


$$\begin{array}{c} \text{BS}\text{A}\;({\text{m}}^{\text{2}})=\text{1}\text{0}.\text{1}\times \frac{W^{\text{2}/\text{3}}}{{\text{1}\text{0}}^{\text{4}}} \end{array}$$ (1)

where *W* is body weight in grams.

The reference flow rate (*Q*_ref_) was determined using Equation 2:


$$\begin{array}{c} Q_{\text{r}\text{ef}}\;(\text{mL}/\text{min})={PI}_{\text{eff}}\times \text{BSA}\times \text{1},\text{000} \end{array}$$ (2)

where *PI*_eff_ is the effective perfusion index (L/min/m^2^).

Veterinary CPB flow requirements vary with body size, temperature, hemodilution, and phase of bypass [4,5]. In a reported 2.4-kg toy-breed dog managed under moderate hypothermia (minimum rectal temperature, 26.6°C; target minimum, 28°C), pump flow was 280 mL/min before aortic cross-clamping and 150–230 mL/min during cross-clamping; the target maximum flow was 370 mL/min [11]. Thus, the 300 mL/min reference used in the present bench model is of the same order as the approximately 280 mL/min flow reported before aortic cross-clamping in a similarly sized 2.4-kg dog under moderate hypothermia, but it should not be interpreted as a universal target across CPB phases. In an earlier series of small and toy-breed dogs undergoing deeper hypothermia (mean lowest esophageal temperature, 21.4°C), the mean lowest pump flow during aortic cross-clamping was 29.2 mL/kg/min [5]. These reports indicate that reported perfusion flows vary substantially with temperature and operative phase and do not define a single universal physiologic upper limit. Accordingly, *PI*_eff_ was prespecified at 1.4 L/min/m^2^ as a pragmatic low-flow reference for the present 3-kg bench model rather than as a universal physiologic or clinical target. For a 3-kg model (BSA = 0.21 m^2^), Equation 2 yielded *Q*_ref_ = 294 mL/min, which was rounded to 300 mL/min as the reference pump flow-rate setting for subsequent testing.

### Theoretical geometric indices

To achieve steady, fully developed laminar flow of a Newtonian fluid in a rigid cylindrical tube, the pressure change (Δ*P*_HP_) was described using the Hagen–Poiseuille law (Equation 3) [15,19].


$$\begin{array}{c} {\Delta P}_{\text{H}\text{P}}=Q_{\text{H}\text{P}}\;\left({\text{cm}}^{\text{3}}/\text{s}\right)\times \text{1}\text{2}\text{8}\;\mu L\;\times \frac{\text{7}.\text{5}\text{0}\text{0}\text{6}\times {\text{1}\text{0}}^{-\text{4}}}{{\pi D}_{\text{i}}^{\text{4}}} \end{array}$$ (3)

where *Q*_HP_ is volumetric flow rate in cm^3^/s (1 cm^3^ = 1 mL), *μ* is dynamic viscosity in P (dyn·s/cm^2^), *L* is the length used for geometric comparison in cm, and *D*_i_ is inner diameter in cm. The factor 7.5006 × 10^−4^ was applied to convert pressure from dyn/cm^2^ to mmHg (1 mmHg = 1333.22 dyn/cm^2^). Under these assumptions, Δ*P*_HP_ scales with *L*/*D*_i_^4^.

Considering the tested cannulas/catheters include localized losses (e.g., entrance/exit and side-hole effects) and the calculated pressure difference reflects the test device plus the tubing between the two pressure-measurement sites, Equation (3) was not used to predict outlet pressure directly. Instead, two normalized geometric indices were calculated from the dimensions in Table 1 and expressed relative to the 8-Fr cannula: (1) the normalized aspect ratio (*L*/*D*_i_)_device_/(*L*/*D*_i_)_8-Fr_ and (2) the normalized diameter-based resistance factor (*D*_i_^-4^)_device_/(*D*_i_^-4^)_8-Fr_. These indices were used as interpretable measures of geometry-driven, distributed friction, distinct from localized loss components. For an order-of-magnitude check based on the nominal pump flow-rate settings, the Reynolds number was expected to remain within the laminar or transitional-low range at settings ≤300 mL/min, supporting the use of the Hagen–Poiseuille relation as a descriptive framework, although localized non-ideal effects at the tip and side holes were still expected.

### Experimental protocol and outcome measures

Before measurements in the mock circuit, roller-pump occlusion was adjusted with the 1/4-inch circuit tubing mounted in the Stockert S5 pump head. Occlusion was checked using a previously described fixed flow rate–injection pressure approach [20], in which fluid was delivered with a syringe pump at 0.50 mL/min and the resulting injection pressure was used as an indicator of the degree of occlusion; the occlusion setting was then kept unchanged throughout testing. For each device, the pump flow-rate setting displayed on the Stockert S5 was increased from 50 to 350 mL/min in 50-mL/min increments. Actual circuit flow was not independently measured using a separate flow meter. Circuit-fluid temperature was monitored using the temperature sensor of the Stockert S5 and maintained at 28°C using the HHC-51 temperature control unit. Before testing, both pressure transducers were zeroed to atmospheric pressure. At each pump flow-rate setting, *P*_in_ was regulated to 60 mmHg by manually adjusting the degree of occlusion of the pressure-regulating tubing clamp while monitoring the *P*_in_ pressure display. Increasing clamp occlusion increased downstream hydraulic resistance and thereby increased *P*_in_, whereas reducing the occlusion decreased *P*_in_. After *P*_in_ had been adjusted to the target value, the circuit was allowed to stabilize for at least 60 s. The target *P*_in_ of 60 mmHg was confirmed, and a single *P*_out_ value was then recorded from the physiologic monitor connected to the *P*_out_ pressure transducer at 28°C.

For the 14-G catheters, two configurations were tested at each effective length (14-G 25, 14-G 32, and 14-G 38): (1) the original design with side holes and (2) a sealed condition in which the side-hole region was occluded by wrapping a short segment of non-elastic tape around the catheter. The 8-Fr arterial cannula was tested first, followed by the 14-G catheters in order of effective length: 25, 32, and 38 mm. At each effective length, the side-hole specimen was tested before the separate specimen assigned to the sealed condition. Within each configuration, the pump flow-rate setting was increased sequentially from 50 to 350 mL/min in 50-mL/min increments, and this full sequence was repeated six times, yielding six technical replicates at each pump flow-rate setting. Because the side-hole and sealed conditions at each effective length were evaluated using separate physical catheters, the measurements were not paired by physical specimen or experimental run. The configuration order was fixed rather than randomized or interleaved. No visible leakage from the sealed side-hole region was observed during steady-state testing. Cyanoacrylate adhesive was used only to seal tubing junctions and was not applied to intraluminal catheter surfaces. The 8-Fr arterial cannula was tested in a single configuration (no side holes). Each combination of pump flow-rate setting, device, and side-hole configuration was repeated six times under steady-state conditions; these observations represent technical replicates obtained using the same physical device and circuit setup and were intended to assess bench repeatability rather than device-to-device variability.

The primary outcome was the outlet pressure at the 300 mL/min pump flow-rate setting of the three side-hole 14-G specimens compared with that of the 8-Fr cannula. Secondary outcomes were outlet pressure across all pump flow-rate settings and outlet-pressure differences between the side-hole and sealed configurations at the 300 mL/min pump flow-rate setting. For descriptive analysis, the pressure change across the measured segment (Δ*P*_seg_) was defined using Equation 4:


$$\begin{array}{c} {\Delta P}_{\text{s}\text{eg}}=P_{\text{o}\text{ut}}-P_{\text{i}\text{n}} \end{array}$$ (4)

where *P*_out_ denotes the recorded pressure measured 1,000 mm upstream of the test device and *P*_in_ denotes the regulated target pressure of 60 mmHg monitored 300 mm downstream of the test device. Trial-level *P*_in_ values were not retained; therefore, Δ*P*_seg_ was calculated using the regulated *P*_in_ target of 60 mmHg.

Apparent hydraulic resistance (*R*_app_) was calculated using Equation 5:


$$\begin{array}{c} R_{\text{a}\text{pp}}=\frac{{\Delta P}_{\text{s}\text{eg}}}{Q_{\text{s}\text{eg}}} \end{array}$$ (5)

where *Q*_seg_ denotes the pump flow-rate setting displayed on the Stockert S5 (mL/min), rather than independently measured circuit flow, and *R*_app_ is expressed in mmHg/(mL/min). Accordingly, *R*_app_ was interpreted as a setting-based apparent hydraulic index within the present mock circuit rather than as hydraulic resistance calculated from independently measured flow.

### Statistical analysis

All statistical analyses were performed in Python (v3.12.12) using NumPy (v2.0.2), pandas (v2.2.2), SciPy (v1.16.3), and statsmodels (v0.14.6). Data are reported as the mean ± SD of six technical replicates per condition. Because the six observations per condition were technical replicates obtained using the same physical device and circuit setup, all inferential analyses were treated as exploratory summaries of within-setup contrasts and repeatability rather than as tests supporting device-level population inference. Accordingly, interpretation emphasized means, SDs, and absolute differences, whereas confidence intervals, *p* values, and standardized effect sizes were treated as exploratory summaries of the technical measurements.

Exploratory one-way analysis of variance (ANOVA) was performed for each pump flow-rate setting (50–350 mL/min) to compare outlet pressure among the four configurations included in the primary device comparison: the 8-Fr cannula and the side-hole 14-G 25, 14-G 32, and 14-G 38 specimens. When the ANOVA outcome was significant (*p* ≤ 0.05), exploratory post-hoc pairwise comparisons versus the 8-Fr cannula were conducted using Welch’s two-sample *t*-test. Partial eta-squared (*η*_p_^2^) is reported as a descriptive standardized measure of between-configuration separation within the present bench dataset. For families of pairwise Welch’s *t*-tests, *p* values were adjusted for multiple comparisons using the Holm method. Families were defined as: (i) device-versus-8-Fr comparisons at the 350 mL/min pump flow-rate setting (three tests), (ii) side-hole comparisons at the 300 mL/min pump flow-rate setting across catheter lengths (three tests), and (iii) exploratory side-hole comparisons across pump flow-rate settings within each catheter length (seven tests per length).

To compare the side-hole and sealed conditions, Welch’s two-sample *t*-test was used at each effective length because the two conditions were evaluated using separate physical catheters and the measurements were not paired by physical specimen or experimental run. Mean differences (side-hole value minus sealed value) and corresponding 95% confidence intervals (CIs) were estimated using Welch’s *t*-based method with Welch–Satterthwaite degrees of freedom. Hedges’ *g* was calculated as a descriptive standardized effect-size measure for each contrast. Because each length-by-configuration condition was represented by a single physical catheter and six technical replicates, these comparisons were intended to characterize differences between the tested specimens and measurement variability within the present bench setup and not to provide device-level population inference regarding the side-hole configuration. All tests were two-tailed, and *p* ≤ 0.05 was used as the nominal significance threshold for the exploratory analyses.

To support reproducibility, trial-level *P*_out_ data underlying the analyses are provided in the S1 File (*n* = 294 observations; 8-Fr arterial cannula and 14-G hemodialysis catheters) and are also archived on Zenodo (https://doi.org/10.5281/zenodo.22293399). A Google Colab–compatible Jupyter notebook that reproduces the analyses and outputs reported in the manuscript is provided in the S2 File.

### Ethics statements

This in vitro bench study used a mock CPB circuit and did not involve human participants or live animals. Therefore, ethics committee approval and informed consent were not required.

## Results

### Outlet pressure across pump flow-rate settings

Fig 2 shows the relationship between pump flow-rate setting and outlet pressure for the 8-Fr arterial cannula and the three side-hole 14-G hemodialysis catheter specimens (14-G 25, 14-G 32, and 14-G 38) in the mock 3-kg small-dog CPB circuit. Across the predefined primary analysis range of 50–300 mL/min, mean outlet pressures increased in a near-linear manner as the pump flow-rate setting increased and remained closely clustered among the four tested configurations at each pump flow-rate setting (Fig 2). Exploratory ANOVA performed separately at each pump flow-rate setting did not identify statistically detectable between-configuration separation over this range (*F*(3, 20) ≤ 1.52, *p* ≥ 0.241, partial *η*_p_^2^ ≤ 0.19). At the exploratory 350 mL/min pump flow-rate setting, mean outlet pressures separated more clearly among configurations, and exploratory ANOVA yielded *F*(3, 20) = 164.87, *p* < 0.001, and partial *η*_p_^2^ = 0.96. Pairwise contrasts versus the 8-Fr cannula at the 350 mL/min pump flow-rate setting are provided in S2 Table. These inferential statistics are presented as exploratory summaries of the technical measurements obtained within the present bench setup. Detailed ANOVA outputs and pairwise estimates are provided in S1 and S2 Tables, respectively.

**Fig 2:**
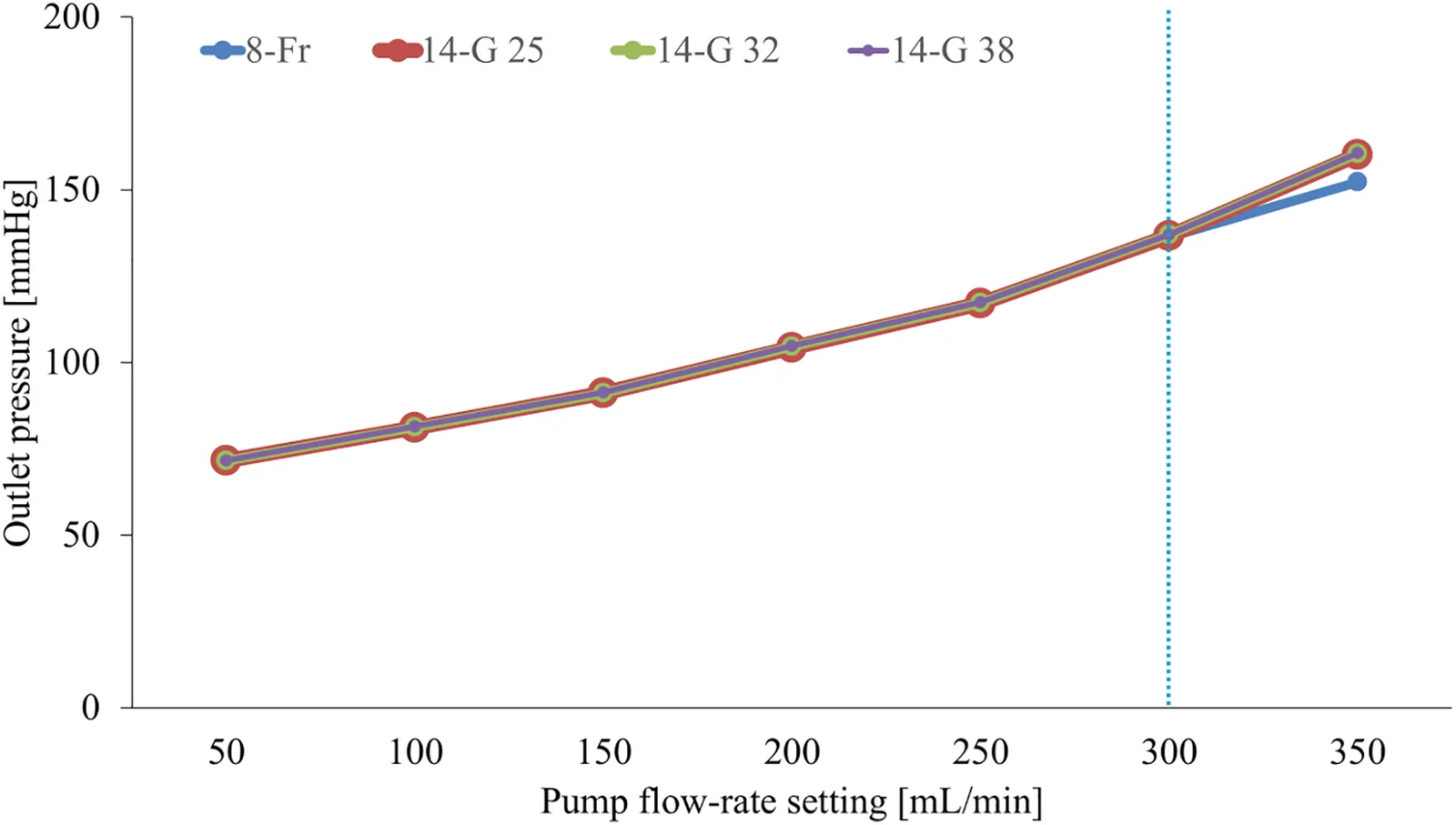
Outlet pressure versus pump flow-rate setting in the mock 3-kg small-dog cardiopulmonary bypass circuit. Outlet pressure (mean ± SD, *n* = 6 technical replicates for each device–pump-setting condition) is shown for the 8-Fr arterial cannula and the side-hole 14-G hemodialysis catheter specimens with effective lengths of 25, 32, and 38 mm. The vertical dotted line indicates 300 mL/min (upper limit of the predefined primary analysis range); 350 mL/min was treated as an exploratory condition above the prespecified primary analysis range. Error bars represent SD and are smaller than the symbols for most data points. SD, standard deviation.

### Primary comparison at the 300 mL/min pump flow-rate setting

Table 2 summarizes the outlet pressure at the 300 mL/min pump flow-rate setting. Mean outlet pressure for the 8-Fr cannula was 136.3 ± 1.21 mmHg, compared with 136.7 ± 1.37, 137.0 ± 1.10, and 137.0 ± 0.89 mmHg for the side-hole 14-G 25, 14-G 32, and 14-G 38 specimens, respectively. The corresponding calculated Δ*P*_seg_, using the regulated *P*_in_ target of 60 mmHg, ranged 76.3–77.0 mmHg, and *R*_app_ was 0.25–0.26 mmHg/(mL/min) for all four primary-comparison configurations. At the 300 mL/min pump flow-rate setting, mean outlet-pressure differences versus the 8-Fr cannula were +0.33 to +0.67 mmHg, with corresponding 95% CIs ranging from −1.33 to +2.15 mmHg. Because no equivalence margin was prespecified, these estimates are reported as descriptive measures of the observed differences and uncertainty within the present bench setup and should not be interpreted as demonstrating formal equivalence.

**Table 2:** Outlet pressure, pressure change, and apparent hydraulic resistance at the 300 mL/min pump flow-rate setting in the 3-kg small-dog cardiopulmonary bypass circuit.

Device	Side hole	Pump flow-rate setting	Outlet pressure	Δ*P*_seg_	*R*_app_	Mean difference vs 8-Fr (95% CI)
	**[yes/no]**	**[mL/min]**	**[mmHg]**	**[mmHg]**	**[mmHg/(mL/min)]**	**[mmHg]**
**8-Fr**	no	300	136.3 ± 1.21	76.3	0.25	0.00 (reference)
**14-G 25**	yes	300	136.7 ± 1.37	76.7	0.26	+0.33 (−1.33, + 2.00)
**14-G 32**	yes	300	137.0 ± 1.10	77.0	0.26	+0.67 (−0.82, + 2.15)
**14-G 38**	yes	300	137.0 ± 0.89	77.0	0.26	+0.67 (−0.72, + 2.05)

Note: Data are expressed as the mean ± SD of six technical replicates. Δ*P*_seg_ was calculated as *P*_out_ − 60 mmHg, where *P*_out_ was recorded 1,000 mm upstream of the test device and 60 mmHg was the regulated *P*_in_ target monitored 300 mm downstream of the test device. Trial-level *P*_in_ values were not retained. *R*_app_ was defined as Δ*P*_seg_ divided by the pump flow-rate setting displayed on the Stockert S5 and expressed in mmHg/(mL/min); it was therefore interpreted as a setting-based apparent hydraulic index rather than as resistance calculated from independently measured circuit flow. Mean differences versus the 8-Fr cannula and corresponding 95% CIs were estimated using Welch’s *t*-based method (Welch–Satterthwaite degrees of freedom). SD, standard deviation; CI, confidence interval

### Outlet-pressure differences between side-hole and sealed configurations at the 300 mL/min pump flow-rate setting

Outlet-pressure differences between the side-hole and sealed configurations at the 300 mL/min pump flow-rate setting are summarized in Table 3. For all effective lengths, catheters with side holes showed lower outlet pressures than those without (sealed configuration) (14-G 25: 136.7 ± 1.37 vs 139.3 ± 1.21 mmHg; 14-G 32: 137.0 ± 1.10 vs 139.8 ± 1.17 mmHg; 14-G 38: 137.0 ± 0.89 vs 139.8 ± 0.75 mmHg). The respective mean differences (side-hole value minus sealed value) were −2.67 mmHg, −2.83 mmHg, and −2.83 mmHg, corresponding to an approximate difference of 2% relative to the sealed configuration. The corresponding 95% CIs and exploratory statistical comparisons are presented in Table 3. Because each length-by-configuration condition was represented by one physical catheter, the comparisons characterize differences between the tested specimens and measurement variability within the present bench setup rather than an isolated side-hole effect or a device-level population effect. A summary of side-hole differences across all tested pump flow-rate settings is provided in S3 Table.

**Table 3:** Outlet pressure with side holes versus the sealed configuration at the 300 mL/min pump flow-rate setting in 14-G catheters.

Device	Outlet pressure with side holes	Outlet pressure without side holes	Mean difference (95% CI)	Holm-adjusted *p*-value	Hedges’ *g*
	**[mmHg]**	**[mmHg]**	**[mmHg]**		
**14-G 25**	136.7 ± 1.37	139.3 ± 1.21	−2.67 (−4.33, −1.00)	0.005	1.91
**14-G 32**	137.0 ± 1.10	139.8 ± 1.17	−2.83 (−4.29, −1.38)	0.003	2.31
**14-G 38**	137.0 ± 0.89	139.8 ± 0.75	−2.83 (−3.90, −1.77)	< 0.001	3.16

Note: Data are expressed as the mean ± standard deviation of six technical replicates per tested specimen/condition. Holm-adjusted *p*-values were obtained using Welch’s two-sample *t*-test comparing catheters with side holes and those without (sealed configuration) at the 300 mL/min pump flow-rate setting, with adjustment across the three prespecified length-specific comparisons. Mean differences were calculated as the with-side-hole value minus the sealed value, and 95% confidence intervals (CIs) were estimated using Welch’s *t*-based method (Welch–Satterthwaite degrees of freedom). Hedges’ *g* is reported as the absolute value as a descriptive standardized summary of the technical measurements and should not be interpreted as a device-level population effect.

### Apparent hydraulic resistance and geometric indices

Across the full pump flow-rate setting range, apparent hydraulic resistance (*R*_app_) showed a shallow U-shaped pattern for the four primary-comparison configurations (Fig 3), with a slight decrease from 50 to 150 mL/min, a near-plateau from 150 to 250 mL/min, and an increase from 300 to 350 mL/min. At each pump flow-rate setting, differences in *R*_app_ among these four configurations were small, with overlapping curves and values clustered around 0.21–0.29 mmHg/(mL/min).

**Fig 3:**
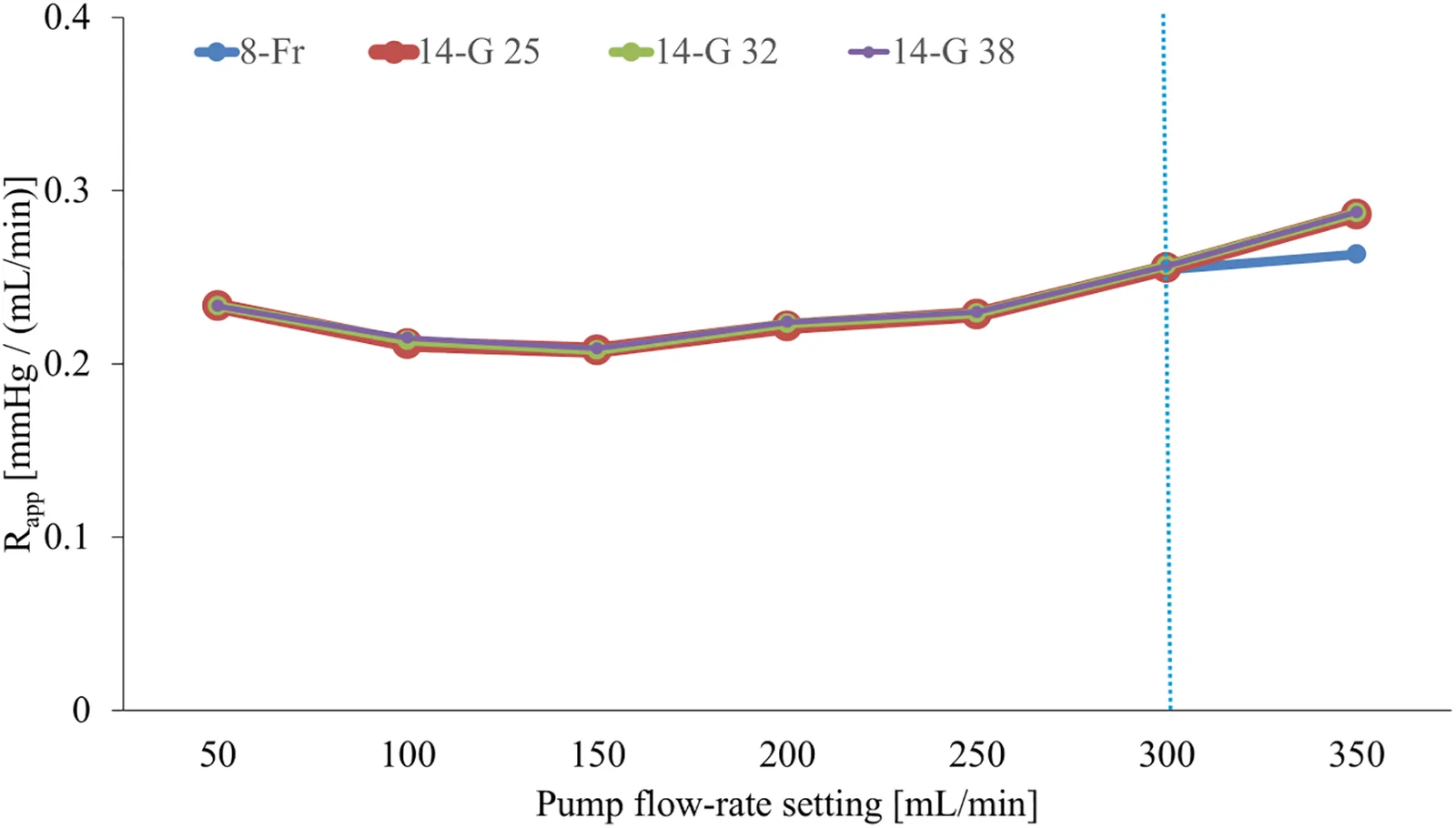
Pump-setting–apparent-resistance relationship in the mock small-dog cardiopulmonary bypass circuit. *R*_app_ (mmHg/[mL/min]) is plotted against the pump flow-rate setting displayed on the Stockert S5 for the 8-Fr arterial cannula and the 14-G hemodialysis catheters with side holes (effective lengths 25, 32, and 38 mm). At each pump flow-rate setting, *R*_app_ was calculated as Δ*P*_seg_/*Q*_seg_, where Δ*P*_seg_ was calculated from the recorded *P*_out_ value and the regulated *P*_in_ target of 60 mmHg, and *Q*_seg_ denotes the pump flow-rate setting displayed on the Stockert S5 rather than independently measured circuit flow. Error bars are omitted because the standard deviations were small relative to the plotted trends. The vertical dotted line at 300 mL/min indicates the upper limit of the predefined primary analysis range; 350 mL/min was treated as an exploratory condition.

Table 4 summarizes geometric indices. Relative to the 8-Fr cannula (*D*_i_ = 2.0 mm), the 14-G catheters had smaller inner diameters (*D*_i_ = 1.6 mm), corresponding to (2.0/1.6)^4^ = 2.44-fold larger diameter-based resistance factor (*D*_i_^-4^). In contrast, the normalized aspect ratio, (*L*/*D*_i_)_device_/(*L*/*D*_i_)_8−Fr_, ranged 0.52–0.79 across effective lengths of 25–38 mm.

**Table 4:** Geometric parameters and theoretical resistance indices for the 8-Fr arterial cannula and 14-G hemodialysis catheters.

Device	Length used for geometric comparison, *L*	Inner diameter, *D*_i_	Normalized *L*/*D*_i_ (relative to 8-Fr)	Theoretical diameter-based resistance factor, *D*_i_^-4^ (relative to 8-Fr)
	**[mm]**	**[mm]**		
**8-Fr**	60	2.0	1.00	1.00
**14-G 25**	25	1.6	0.52	2.44
**14-G 32**	32	1.6	0.67	2.44
**14-G 38**	38	1.6	0.79	2.44

Note: The normalized aspect ratio was calculated as (*L*/*D*_i_)_device_/(*L*/*D*_i_)_8−Fr_. The diameter-based resistance factor (*D*_i_^-4^) was derived from the Hagen–Poiseuille scaling (resistance ∝ *D*_i_^-4^) using the 8-Fr cannula (*D*_i_ = 2.0 mm) as the reference (factor = 1.00). These indices are provided as descriptive geometric indicators and were not used to predict measured outlet pressure.

Despite these theoretical differences in diameter-based indices, mean outlet-pressure differences for the three side-hole 14-G specimens versus the 8-Fr cannula at the 300 mL/min pump flow-rate setting were +0.33 to +0.67 mmHg, and *R*_app_ values were 0.25–0.26 mmHg/(mL/min) across these four tested configurations (Table 2; Figs 2 and 3).

## Discussion

### Summary of main findings

This in vitro bench study evaluated one 8-Fr pediatric arterial cannula and six 14-G hemodialysis catheter specimens. The primary device comparison included the 8-Fr cannula and the three side-hole 14-G specimens (14-G 25, 14-G 32, and 14-G 38), whereas separate sealed specimens were evaluated for the side-hole comparison. Across the predefined primary analysis range (50–300 mL/min), outlet pressure increased in a near-linear manner as the pump flow-rate setting increased and remained closely clustered among devices at each pump flow-rate setting (Fig 2). At the prespecified reference pump flow-rate setting of 300 mL/min, mean outlet-pressure differences versus the 8-Fr cannula were +0.33 to +0.67 mmHg, with corresponding 95% CIs ranging from −1.33 to +2.15 mmHg (Table 2). These estimates describe the magnitude and uncertainty of the observed bench-level differences and do not establish formal equivalence.

Across the 50–300 mL/min pump flow-rate setting range, mean outlet pressures remained closely clustered among the tested configurations, and exploratory ANOVA performed separately at each pump flow-rate setting did not identify statistically detectable between-configuration separation (S1 Table). At the exploratory 350 mL/min pump flow-rate setting, a clearer separation in mean outlet pressure was observed, with all three side-hole 14-G specimens showing higher values than the 8-Fr cannula (S2 Table). These statistical comparisons characterize the present technical measurements and should not be interpreted as device-level population inference.

A modest but consistent difference between the side-hole and sealed configurations was observed at the 300 mL/min pump flow-rate setting: outlet pressure was 2.7–2.8 mmHg lower with side holes than with the sealed configuration (Table 3), corresponding to an approximate difference of 2%. Although the 14-G catheters have a substantially higher theoretical diameter-based resistance factor (Table 4), the observed mean outlet-pressure differences versus the 8-Fr cannula were ≤0.67 mmHg at the 300 mL/min pump flow-rate setting under the present bench conditions. The *R*_app_ values were likewise tightly clustered across devices.

### Hydraulic determinants and comparison with theory

Hydraulic resistance in small-diameter perfusion devices is governed primarily by inner diameter, effective length, and local tip/side-hole geometry. Under steady, fully developed laminar conditions in a rigid cylindrical tube, the Hagen–Poiseuille relation predicts that Δ*P*_HP_ scales with *L*/*D*_i_^4^ [19], providing a theoretical basis for comparing geometry-driven pressure losses among devices. In the present devices, *D*_i_ was 1.6 mm for the 14-G catheters and 2.0 mm for the 8-Fr cannula, corresponding to a diameter ratio of 1.25 and an expected relative diameter-based resistance factor of approximately 2.4 (Table 4).

Despite this theoretical contrast, outlet pressure at the 300 mL/min pump flow-rate setting differed by < 1 mmHg among devices (Table 2), and *R*_app_ values were tightly clustered (Fig 3). This apparent discrepancy is consistent with the study design. The calculated pressure difference, Δ*P*_seg_, reflects the combined hydraulic behavior of the test device and the tubing between the two pressure-measurement sites (1,000 mm upstream and 300 mm downstream of the test device), and the tested effective lengths (25–38 mm) are short relative to this measured circuit segment. Under such conditions, localized losses and circuit-level contributions can meaningfully influence the observed pressure response across pump flow-rate settings, limiting the extent to which diameter-based scaling alone predicts observed differences [3].

Across the full pump flow-rate setting range, *R*_app_ showed a shallow U-shaped profile (Fig 3). This behavior is compatible with predominantly laminar flow in short segments, where small changes in measurement noise, entrance effects, minor pump pulsatility, and local geometry can affect apparent resistance estimates derived from Δ*P*_seg_/*Q*_seg_ [19]. Thus, these results should be interpreted as circuit-level hydraulic comparisons rather than intrinsic measurements of device resistance alone.

### Outlet-pressure differences between side-hole configurations

At the 300 mL/min pump flow-rate setting, outlet pressure was approximately 2.7–2.8 mmHg lower with side holes than with the sealed configuration across the three tested effective lengths (Table 3), corresponding to an absolute difference of approximately 2%. A possible explanation is redistribution of flow between the distal opening and side holes, which may alter local flow structures near the catheter tip [12,13]. However, local velocity fields, flow partitioning among individual openings, and near-tip pressure distributions were not measured directly in the present study; therefore, this explanation should be regarded as a mechanistic hypothesis rather than a directly demonstrated mechanism. Because the side-hole and sealed conditions at each effective length were tested using separate physical catheters, the observed differences may reflect both configuration-related effects and specimen-specific variation. Accordingly, these findings should be interpreted as descriptive contrasts between the tested specimens under the present bench conditions rather than as an isolated causal effect of the side holes or as a device-level population effect.

### Relevance to small-dog cardiopulmonary bypass

The circuit was scaled to emulate CPB conditions expected for a 3-kg small dog—a clinically relevant scenario in veterinary cardiac surgery where arterial access is constrained by small vessel calibers and dedicated cannulas may be limited [4–6]. Within the prespecified 50–300 mL/min pump flow-rate setting range, the three side-hole 14-G specimens produced outlet pressure and *R*_app_ values with only small observed differences compared with those of the 8-Fr cannula in this mock circuit. However, because the present experiments used technical replicates in a bench model, these data should be interpreted as circuit-level hydraulic benchmarks within the standardized circuit and not as establishing clinical interchangeability or device-to-device equivalence [4,5,11].

Because the absolute outlet-pressure values in this study depend on the specific mock-circuit configuration and pressure-measurement location, they should be interpreted only as standardized circuit-level hydraulic measurements and not as evidence of clinically acceptable or safe pressure levels.

More broadly, the geometric–hydraulic relationships examined herein may inform device selection and circuit optimization in other low-body-mass perfusion settings. This includes neonatal/low-prime circuit concepts and miniature CPB applications, provided that scaling and validation are performed for the target context [7–9]. Evidence from previous cases applying CPB in miniature neonates further supports the relevance of low-prime, low-body-mass perfusion constraints [21].

### Limitations and future directions

First, the experiments were performed using a Newtonian glycerol–water solution to isolate geometric and hydraulic effects [14,22]. Therefore, the non-Newtonian rheology of blood, its cellular components, and hemolysis were not evaluated. Accordingly, any statements regarding blood damage are limited to the hydraulic context only and do not constitute evidence of hemocompatibility; dedicated ex vivo or in vivo studies using blood are required to establish this point [1,2,23].

Second, the mock circuit lacked vascular compliance, pulsatility, and autoregulation, and pressures were measured 300 mm downstream and 1,000 mm upstream rather than immediately adjacent to the test device. This configuration was chosen to represent arterial-line pressure monitoring in the mock CPB circuit and to capture the combined hydraulic behavior of the test device and the tubing between the two pressure-measurement sites [4–6,9]. Consequently, the absolute values should be interpreted as standardized comparisons within the measured circuit segment rather than as direct surrogates for in vivo arterial pressure at the device tip. In addition, although *P*_in_ was adjusted to and confirmed at the 60 mmHg target before *P*_out_ recording, trial-level *P*_in_ values were not retained. Accordingly, Δ*P*_seg_ and *R*_app_ were calculated using the regulated *P*_in_ target of 60 mmHg rather than trial-level recorded *P*_in_ values. Actual circuit flow was not independently measured with a separate flow meter; experimental flow conditions were defined by the pump flow-rate settings displayed on the Stockert S5. Therefore, analyses involving flow, including *R*_app_, should be interpreted as setting-based bench comparisons rather than measurements based on independently verified circuit flow.

Third, six replicates per condition represented technical replicates obtained using the same physical device and circuit setup; manufacturing variability and between-animal anatomical variability were not assessed. Accordingly, the reported confidence intervals, *p* values, and standardized effect sizes should be interpreted as exploratory summaries of contrasts and repeatability within the present bench setup and not as device-to-device, device-type population, or animal-level inference. Multiple independent physical specimens of each device type would be required to support population-level inference regarding device performance. Future research should therefore include multiple samples per device type and consider formal equivalence testing where the goal is to conclude practical similarity within predefined margins [24].

Fourth, only one physical 14-G catheter was tested for each combination of effective length and side-hole configuration, and the side-hole and sealed conditions at a given effective length were evaluated using separate physical catheters. In addition, testing followed a fixed sequence: the 8-Fr cannula was tested first, followed by the 14-G catheters at effective lengths of 25, 32, and 38 mm, with the side-hole specimen tested before the sealed specimen at each length. The conditions were not randomized or interleaved. Therefore, the observed differences between side-hole configurations cannot be separated completely from specimen-specific variation or possible order-related or other time-dependent influences. These findings should not be interpreted as an isolated causal effect of the side holes. Future studies should include multiple independent physical specimens for each length and configuration and use randomized or counterbalanced experimental sequences where technically feasible.

Fifth, the sealed condition was created by external taping to occlude the side holes, which may have altered local stiffness or outer geometry. Purpose-built catheters without side holes, flow visualization, or both would better isolate the contribution of side-hole outflow [12,13].

Finally, this study focused on hydraulic performance and did not evaluate procedural and clinical endpoints (e.g., cannulation difficulty, arterial injury). Such outcomes must be integrated with the present hydraulic benchmarks to validate 14-G catheters as arterial access devices in small-dog CPB [25].

## Conclusions

This in vitro bench study provides circuit-level hydraulic reference data for one 8-Fr pediatric arterial cannula and six 14-G hemodialysis catheter specimens at effective lengths of 25, 32, and 38 mm, with separate side-hole and sealed specimens at each length. Across the prespecified 50–300 mL/min pump flow-rate setting range, outlet-pressure differences between the three side-hole 14-G specimens and the 8-Fr cannula were small under the present bench conditions; at the 300 mL/min pump flow-rate setting, mean differences versus the 8-Fr cannula were +0.33 to +0.67 mmHg. Among the tested 14-G specimens, outlet pressure was 2.7–2.8 mmHg lower for the side-hole specimens than for the sealed specimens at 300 mL/min. At the exploratory 350 mL/min pump flow-rate setting, all three side-hole 14-G specimens showed higher outlet pressures than the 8-Fr cannula. These technical-replicate findings do not establish device-level equivalence, interchangeability, or clinical validity. Future studies should evaluate multiple independent physical specimens and clinically relevant validation models, with formal equivalence testing where predefined device-level similarity is the intended conclusion.

## Supporting information

S1 Table Exploratory one-way ANOVA of outlet pressure at each pump flow-rate setting among the 8-Fr cannula and three side-hole 14-G specimens in the mock 3-kg small-dog cardiopulmonary bypass circuit. Note: Data comprise one-way ANOVA results at each pump flow-rate setting, comparing outlet pressure among the 8-Fr arterial cannula and three side-hole 14-G hemodialysis catheter specimens (14-G 25, 14-G 32, and 14-G 38). *N* denotes the total number of technical measurements (six technical replicates for each of four tested configurations) and does not represent independent physical devices. ANOVA, one-way analysis of variance.
(DOCX)

S2 Table Exploratory post-hoc comparisons of outlet pressure at the 350 mL/min pump flow-rate setting between three side-hole 14-G specimens and the 8-Fr arterial cannula. Note: Pairwise Welch’s *t*-tests comparing each side-hole 14-G specimen (14-G 25, 14-G 32, and 14-G 38) with the 8-Fr arterial cannula at the 350 mL/min pump flow-rate setting. Data are expressed as mean ± standard deviation (*n* = 6 technical replicates per tested specimen). Hedges’ *g* is reported as the absolute value as a descriptive standardized summary of the technical measurements and should not be interpreted as a device-level population effect. Holm-adjusted *p* values are reported for the three prespecified comparisons.
(DOCX)

S3 Table Outlet-pressure differences between side-hole and sealed configurations across all pump flow-rate settings in 14-G hemodialysis catheters. Note: Data are expressed as mean ± standard deviation (*n* = 6 technical replicates per tested specimen/configuration). Mean difference is calculated as (with side holes − without side holes). Holm-adjusted *p* values were obtained using Welch’s *t*-tests comparing catheters, with adjustment applied within each effective length across the seven pump flow-rate settings. Hedges’ *g* is reported as the absolute value as a descriptive standardized summary of the technical measurements and should not be interpreted as a device-level population effect.
(DOCX)

S1 File Trial-level dataset. Trial-level *P*_out_ observations underlying the analyses (*n* = 294 observations; 8-Fr arterial cannula and 14-G hemodialysis catheters).
(CSV)

S2 File Analysis notebook. Google Colab–compatible Jupyter notebook reproducing the analyses and outputs reported in the manuscript (IPYNB).
(IPYNB)
