# Supplementary material for: Hydraulic performance of 14-G hemodialysis catheters versus an 8-Fr arterial cannula in a mock 3-kg small-dog cardiopulmonary bypass circuit
Source: PLoS One. 2026 Oct 26;21(10):e0361473. doi: 10.1371/journal.pone.0361473 (PMC13704862; doi:10.1371/journal.pone.0361473)
Supplement: Supplementary file 1 [file pone.0361473.s001.docx]

**S1 Table. Exploratory one-way ANOVA of outlet pressure at each pump flow-rate setting among the 8-Fr cannula and three side-hole 14-G specimens in the mock 3-kg small-dog cardiopulmonary bypass circuit.**

| **Pump flow-rate setting [mL/min]** | ***k*  (configurations)** | ***N*  (observations)** | ***F*(df1, df2)** | ***p*-value** | ***η*_p_^2^** |
| --- | --- | --- | --- | --- | --- |
| 50 | 4 | 24 | *F*(3, 20) = 0.00 | 1.000 | 0 |
| 100 | 4 | 24 | *F*(3, 20) = 1.52 | 0.241 | 0.19 |
| 150 | 4 | 24 | *F*(3, 20) = 0.44 | 0.724 | 0.06 |
| 200 | 4 | 24 | *F*(3, 20) = 0.90 | 0.458 | 0.12 |
| 250 | 4 | 24 | *F*(3, 20) = 1.14 | 0.356 | 0.15 |
| 300 | 4 | 24 | *F*(3, 20) = 0.46 | 0.714 | 0.06 |
| 350 | 4 | 24 | *F*(3, 20) = 164.87 | < 0.001 | 0.96 |

Note: Data comprise one-way ANOVA results at each pump flow-rate setting, comparing outlet pressure among the 8-Fr arterial cannula and three side-hole 14-G hemodialysis catheter specimens (14-G 25, 14-G 32, and 14-G 38). *N* denotes the total number of technical measurements (six technical replicates for each of four tested configurations) and does not represent independent physical devices.

ANOVA, one-way analysis of variance.
