# Supplementary material for: Hydraulic performance of 14-G hemodialysis catheters versus an 8-Fr arterial cannula in a mock 3-kg small-dog cardiopulmonary bypass circuit
Source: PLoS One. 2026 Oct 26;21(10):e0361473. doi: 10.1371/journal.pone.0361473 (PMC13704862; doi:10.1371/journal.pone.0361473)
Supplement: Supplementary file 2 [file pone.0361473.s002.docx]

**S2 Table. Exploratory post-hoc comparisons of outlet pressure at the 350 mL/min pump flow-rate setting between three side-hole 14-G specimens and the 8-Fr arterial cannula.**

| Comparison | Pump flow-rate setting | Outlet pressure, 8-Fr | Outlet pressure, 14-G | Holm-adjusted *p*-value | Hedges’ *g* |
| --- | --- | --- | --- | --- | --- |
|  | [mL/min] | [mmHg] | [mmHg] |  |  |
| 14-G 25 vs 8-Fr | 350 | 152.17 ± 0.75 | 160.17 ± 0.98 | < 0.001 | 8.43 |
| 14-G 32 vs 8-Fr | 350 | 152.17 ± 0.75 | 160.50 ± 0.84 | < 0.001 | 9.67 |
| 14-G 38 vs 8-Fr | 350 | 152.17 ± 0.75 | 160.67 ± 0.52 | < 0.001 | 12.16 |

Note: Pairwise Welch’s *t*-tests comparing each side-hole 14-G specimen (14-G 25, 14-G 32, and 14-G 38) with the 8-Fr arterial cannula at the 350 mL/min pump flow-rate setting. Data are expressed as mean ± standard deviation (*n* = 6 technical replicates per tested specimen). Hedges’ *g* is reported as the absolute value as a descriptive standardized summary of the technical measurements and should not be interpreted as a device-level population effect. Holm-adjusted *p* values are reported for the three prespecified comparisons.
