# Supplementary material for: Hydraulic performance of 14-G hemodialysis catheters versus an 8-Fr arterial cannula in a mock 3-kg small-dog cardiopulmonary bypass circuit
Source: PLoS One. 2026 Oct 26;21(10):e0361473. doi: 10.1371/journal.pone.0361473 (PMC13704862; doi:10.1371/journal.pone.0361473)
Supplement: Supplementary file 3 [file pone.0361473.s003.docx]

**S3 Table. Outlet-pressure differences between side-hole and sealed configurations across all pump flow-rate settings in 14-G hemodialysis catheters.**

| **Device** | **Pump flow-rate setting** | **Outlet pressure  with side holes** | **Outlet pressure  without side holes** | **Mean difference (95% CI)** | **Holm-adjusted *p*-value** | **Hedges’ *g*** |
| --- | --- | --- | --- | --- | --- | --- |
|  | **[mL/min]** | **[mmHg]** | **[mmHg]** | **[mmHg]** |  |  |
| 14-G 25 | 50 | 71.7 ± 0.52 | 74.8 ± 0.41 | −3.17 (−3.77, −2.56) | < 0.001 | 6.28 |
|  | 100 | 81.2 ± 0.41 | 84.3 ± 0.52 | −3.17 (−3.77, −2.56) | < 0.001 | 6.28 |
|  | 150 | 91.2 ± 0.41 | 94.5 ± 0.55 | −3.33 (−3.96, −2.70) | < 0.001 | 6.37 |
|  | 200 | 104.3 ± 0.52 | 107.5 ± 1.22 | −3.17 (−4.46, −1.87) | 0.002 | 3.11 |
|  | 250 | 117.2 ± 0.75 | 119.5 ± 0.55 | −2.33 (−3.19, −1.48) | < 0.001 | 3.27 |
|  | 300 | 136.7 ± 1.37 | 139.3 ± 1.21 | −2.67 (−4.33, −1.00) | 0.010 | 1.91 |
|  | 350 | 160.2 ± 0.98 | 161.2 ± 0.41 | −1.00 (−2.04, 0.04) | 0.057 | 1.23 |
| 14-G 32 | 50 | 71.7 ± 0.52 | 74.8 ± 0.41 | −3.17 (−3.77, −2.56) | < 0.001 | 6.28 |
|  | 100 | 81.3 ± 0.52 | 84.5 ± 0.55 | −3.17 (−3.85, −2.48) | < 0.001 | 5.49 |
|  | 150 | 91.2 ± 0.41 | 94.7 ± 0.52 | −3.50 (−4.10, −2.90) | < 0.001 | 6.94 |
|  | 200 | 104.7 ± 1.37 | 107.5 ± 1.22 | −2.83 (−4.51, −1.16) | 0.007 | 2.02 |
|  | 250 | 117.3 ± 0.52 | 119.7 ± 1.03 | −2.33 (−3.44, −1.23) | 0.006 | 2.64 |
|  | 300 | 137.0 ± 1.10 | 139.8 ± 1.17 | −2.83 (−4.29, −1.38) | 0.006 | 2.31 |
|  | 350 | 160.5 ± 0.84 | 161.3 ± 0.82 | −0.83 (−1.90, 0.23) | 0.111 | 0.93 |
| 14-G 38 | 50 | 71.7 ± 0.52 | 74.8 ± 0.41 | −3.17 (−3.77, −2.56) | < 0.001 | 6.28 |
|  | 100 | 81.5 ± 0.55 | 84.5 ± 0.55 | −3.00 (−3.70, −2.30) | < 0.001 | 5.06 |
|  | 150 | 91.3 ± 0.52 | 94.8 ± 0.41 | −3.50 (−4.10, −2.90) | < 0.001 | 6.94 |
|  | 200 | 104.8 ± 0.41 | 107.7 ± 0.52 | −2.83 (−3.44, −2.23) | < 0.001 | 5.62 |
|  | 250 | 117.5 ± 0.55 | 120.7 ± 0.52 | −3.17 (−3.85, −2.48) | < 0.001 | 5.49 |
|  | 300 | 137.0 ± 0.89 | 139.8 ± 0.75 | −2.83 (−3.90, −1.77) | < 0.001 | 3.16 |
|  | 350 | 160.7 ± 0.52 | 161.2 ± 0.41 | −0.50 (−1.10, 0.10) | 0.094 | 0.99 |

Note: Data are expressed as mean ± standard deviation (*n* = 6 technical replicates per tested specimen/configuration). Mean difference is calculated as (with side holes − without side holes). Holm-adjusted *p* values were obtained using Welch’s *t*-tests comparing catheters, with adjustment applied within each effective length across the seven pump flow-rate settings. Hedges’ *g* is reported as the absolute value as a descriptive standardized summary of the technical measurements and should not be interpreted as a device-level population effect.
