# Supplementary material for: Hydraulic performance of 14-G hemodialysis catheters versus an 8-Fr arterial cannula in a mock 3-kg small-dog cardiopulmonary bypass circuit
Source: PLoS One. 2026 Oct 26;21(10):e0361473. doi: 10.1371/journal.pone.0361473 (PMC13704862; doi:10.1371/journal.pone.0361473)
Supplement: Supplementary file 5 [file pone.0361473.s007.docx]

**Response to Reviewers**

**PONE-D-26-17814**

Hydraulic performance of 14-G hemodialysis catheters versus an 8-Fr arterial cannula in a mock 3-kg small-dog cardiopulmonary bypass circuit

# Opening statement

We thank the Academic Editor and the reviewers for their careful evaluation of our manuscript. The Academic Editor requested that we address all reviewer comments, with particular attention to improving the statistical analysis and clarifying the flow conditions, inlet pressure, and bypass circuit. Reviewer #1 regarded the study as potentially useful as a standardized bench comparison but raised important concerns regarding the clinical rationale for the comparator, interpretation of statistical inference from technical replicates, the side-hole analysis, and the clinical framing of the flow and pressure findings. Reviewer #2 considered the manuscript technically sound and requested additional clarification of device flow specifications, the mock-circuit configuration and flow direction, device position, and the Conclusions. Detailed point-by-point responses are organized below.

# Summary of major revisions

In response to the Academic Editor and reviewers, we made substantial revisions to the statistical interpretation, experimental description, figures, and clinical framing of the manuscript. First, because the six observations per condition were technical replicates obtained using the same physical device and circuit setup, we reframed all inferential analyses as exploratory summaries of within-setup contrasts and repeatability, placed greater emphasis on descriptive estimates and absolute pressure differences, clarified the multiple-comparison procedures, and corrected the Holm-adjusted *p* values for the side-hole comparisons. Second, we clarified the clinical rationale for using the 8-Fr pediatric arterial cannula as the benchmark comparator and explained why the 6-Fr cannula was not experimentally tested. Third, we expanded the description of the mock CPB circuit and flow conditions. Experimental flow conditions are now explicitly described as the pump flow-rate settings displayed on the Stockert S5. Before measurements, roller-pump occlusion was adjusted and checked using the previously described fixed flow rate–injection pressure approach [20] with a syringe-pump injection flow of 0.50 mL/min, and the occlusion setting was then kept unchanged throughout testing. Actual circuit flow was not independently measured using a separate flow meter. We therefore consistently describe the experimental conditions as pump flow-rate settings and interpret *R*_app_ as a setting-based apparent hydraulic index rather than as resistance calculated from independently measured flow. We also clarified the clinical context for the 300 mL/min reference pump flow-rate setting, designated 50–300 mL/min as the prespecified primary analysis range and 350 mL/min as an exploratory setting, and clarified the *P*_in_ and *P*_out_ measurement sites, use of two pressure transducers, atmospheric zeroing, regulation and confirmation of the 60 mmHg *P*_in_ target before recording a single *P*_out_ value after stabilization, clamp functions, direction of perfusate flow, and approximately 10-mm insertion depth of the test devices. Figure 1 was revised accordingly. Fourth, manufacturer-reported flow information was added to Table 1, with clarification that the available documentation did not specify a maximum recommended blood-flow rate for either tested device. Fifth, we clarified that the side-hole and sealed conditions at each effective length were evaluated using separate physical catheters and that testing followed a fixed, non-randomized sequence (8-Fr first, followed by 14-G catheters at 25, 32, and 38 mm, with the side-hole specimen tested before the sealed specimen at each length). We emphasized the observed absolute pressure differences rather than standardized effect sizes and added both specimen-specific variation and possible order-related influences as limitations. We also removed the potentially misleading numerical comparison with hemodialysis access pressures, clarified that the glycerol–water solution was only an approximate Newtonian blood analogue, and revised the Conclusions to provide a more concise summary of the principal circuit-level findings and clearer directions for future research.

# Response to the Academic Editor

| **Reviewer comment** | The authors are suggested to consider all the comments of reviewers and incorporate recommendations such as improving the statistical analysis and including details about the flow conditions (Flow rate, inlet pressure, bypass circuit) in the revised paper. |
| --- | --- |
| **Response** | We thank the Academic Editor for the careful evaluation and guidance. We addressed all comments raised by both reviewers and revised the manuscript extensively, with particular attention to the statistical interpretation and to clarification of the flow conditions, inlet pressure, and mock bypass circuit.  Regarding the statistical analysis, we clarified that the six observations per condition were technical replicates obtained using the same physical device and circuit setup. Accordingly, ANOVA, Welch’s *t*-tests, confidence intervals, and standardized effect sizes are now explicitly treated as exploratory summaries of within-setup contrasts and repeatability rather than as analyses supporting device-level population inference. We placed greater emphasis on descriptive estimates, standard deviations, and absolute pressure differences, clarified the multiple-comparison families, and corrected the Holm-adjusted *p* values for the side-hole comparisons.  Regarding the flow conditions, we now explicitly state that the experimental conditions were defined by the pump flow-rate settings displayed on the Stockert S5, which were increased from 50 to 350 mL/min in 50-mL/min increments. Before measurements, roller-pump occlusion was adjusted and checked using the previously described fixed flow rate–injection pressure approach [20] with a syringe-pump injection flow of 0.50 mL/min, and the occlusion setting was then kept unchanged throughout testing. Actual circuit flow was not independently measured using a separate flow meter. Accordingly, we revised the manuscript consistently to describe these conditions as pump flow-rate settings and to interpret *R*_app_ as a setting-based apparent hydraulic index rather than as resistance calculated from independently measured flow. We also clarified that 50–300 mL/min was the prespecified primary analysis range, whereas 350 mL/min was an exploratory setting, and that the perfusion index used to derive the 300 mL/min reference was a pragmatic low-flow reference for the present 3-kg bench model rather than a universal physiologic or clinical target.  Regarding inlet pressure and the mock CPB circuit, we clarified that *P*_in_ was monitored 300 mm downstream of the test device and regulated to 60 mmHg by manually adjusting a pressure-regulating tubing clamp located downstream of the *P*_in_ measurement site, whereas *P*_out_ was recorded 1,000 mm upstream of the test device. Two pressure transducers were used and were zeroed to atmospheric pressure before testing. At each pump flow-rate setting, after *P*_in_ had been adjusted to the 60 mmHg target, the circuit was allowed to stabilize for at least 60 s; the target *P*_in_ was then confirmed and a single *P*_out_ value was recorded. Trial-level *P*_in_ values were not retained, and Δ*P*_seg_ and *R*_app_ were therefore calculated using the regulated *P*_in_ target of 60 mmHg. We distinguished the pressure-regulating clamp from a separate circuit-closure clamp that was used only to maintain the closed-loop configuration. We also corrected the description of the test-device position to specify an approximately 10-mm insertion depth into the arterial-line tubing and revised Figure 1 to show the direction of perfusate flow, pressure-measurement sites, clamp functions, and overall circuit configuration.  In addition, we addressed the reviewers’ concerns regarding comparator selection, manufacturer-reported device flow information, interpretation of the side-hole experiment, the blood-analogue viscosity, clinical interpretation of absolute pressure values, and the Conclusions. These revisions were made throughout the Abstract, Introduction, Materials and Methods, Results, Discussion, Conclusions, tables, figures, and Supporting Information. |
| **Manuscript changes** | We revised the manuscript throughout to incorporate the Academic Editor’s recommendations and all reviewer comments. The principal changes included: (1) reframing the statistical analyses as exploratory summaries appropriate to the technical-replicate design and emphasizing descriptive estimates and absolute differences; (2) clarifying that experimental flow conditions were defined by the Stockert S5 pump flow-rate settings, documenting roller-pump occlusion adjustment and fixed flow rate–injection pressure verification at 0.50 mL/min, adding the corresponding method citation as Reference 20, stating that actual circuit flow was not independently measured with a separate flow meter, defining *R*_app_ as a setting-based apparent hydraulic index, and clarifying the prespecified 50–300 mL/min primary analysis range, the clinical context for the 300 mL/min reference pump flow-rate setting, and the exploratory status of the 350 mL/min setting; (3) expanding the description of the mock CPB circuit, including the *P*_in_ and *P*_out_ measurement locations, use and atmospheric zeroing of two pressure transducers, regulation and confirmation of *P*_in_ at 60 mmHg, stabilization for at least 60 s before recording a single *P*_out_ value, non-retention of trial-level *P*_in_ values, the distinct function of the circuit-closure clamp, test-device insertion depth, and direction of perfusate flow; (4) revising Figure 1 accordingly; (5) clarifying the rationale for the 8-Fr comparator and the non-testing of the 6-Fr cannula; (6) adding manufacturer-reported flow information to Table 1; (7) clarifying that separate physical 14-G catheters were used for the side-hole and sealed conditions at each effective length, documenting the fixed experimental sequence, and narrowing the scope of statistical inference; (8) correcting the Holm-adjusted *p* values in Table 3; (9) removing the hemodialysis pressure comparison and limiting interpretation of absolute circuit pressures; (10) clarifying the use of the glycerol–water solution as an approximate Newtonian blood analogue; and (11) streamlining the Conclusions and specifying priorities for future validation and formal equivalence testing. |

# Response to Reviewer #1

**Overall response to Reviewer #1**

We thank Reviewer #1 for the detailed and constructive evaluation. The reviewer recognized the practical relevance and clear presentation of this in vitro bench study while identifying several issues that require careful revision, particularly the scope of statistical inference from technical replicates, the emphasis placed on non-significant hypothesis tests, the side-hole analysis, the clinical rationale for the comparator and reference pump flow-rate setting, and the interpretation of circuit pressures. Each concern is addressed individually below.

**Major Revision**

| **Reviewer comment** | 1. Clinical rationale for selection of the 8-Fr cannula as the comparator. The clinical motivation for using a 14-G hemodialysis catheter is the limited arterial caliber in toy-breed dogs. However, the outer diameter of the 14-G catheter is 2.1 mm, which is substantially closer to that of the 6-Fr cannula (2.0 mm) than to that of the 8-Fr cannula (2.7 mm). The 6-Fr cannula is listed in Table 1 only as a reference and was not experimentally evaluated. Please clarify why the 8-Fr cannula was selected as the primary comparator and why the 6-Fr cannula was not tested. If the reason is that a 6-Fr cannula is expected to produce excessive resistance or cannot provide the required flow, this clinical rationale should be stated explicitly. I do not necessarily consider additional testing of the 6-Fr cannula mandatory, but the choice of comparator requires clearer justification. |
| --- | --- |
| **Response** | We thank the reviewer for this important comment. We agree that the outer diameter of the 14-G catheter (2.1 mm) is geometrically closer to that of the 6-Fr cannula (2.0 mm) than to that of the 8-Fr cannula (2.7 mm). The 6-Fr cannula was included in Table 1 only as a dimensional reference. It was not excluded because of an a priori assumption that it would produce excessive resistance or fail to provide the required flow. Rather, the 6-Fr cannula could not be procured as a new device for standardized experimental testing in Japan [11].  In contrast, the 8-Fr pediatric arterial cannula has been used as an arterial access device in very small dogs and therefore represented a clinically relevant benchmark comparator for the present bench study [11]. We revised the Introduction to explain this rationale explicitly and revised the Table 1 note to clarify that the 6-Fr cannula was included only as a specification reference because a new device could not be procured for standardized experimental testing in Japan. |
| **Manuscript changes** | We revised the Introduction and the Table 1 note to clarify why the 8-Fr cannula was selected as the experimental benchmark comparator and why the 6-Fr cannula was not tested.  **Revised text in the Introduction:**  “Although the 6-Fr arterial cannula (outer diameter, 2.0 mm) is geometrically closer to the 14-G catheter (outer diameter, 2.1 mm), the 6-Fr cannula could not be procured as a new device for standardized experimental testing in Japan [11]. In contrast, the 8-Fr pediatric arterial cannula has been used as an arterial access device in very small dogs and therefore served as a clinically relevant benchmark comparator in the present study [11].”  **Revised Table 1 note:**  “The 6-Fr cannula is included for specification reference only and was not used for experimental testing because it could not be procured as a new device for standardized bench testing in Japan [11].” |

| **Reviewer comment** | 2. Interpretation of statistical inference from technical replicates. The manuscript appropriately states that the six observations per condition are technical replicates obtained using the same physical device and circuit setup rather than six independent devices. Nevertheless, ANOVA, Welch's *t*-tests, confidence intervals, and standardized effect sizes are used extensively. These analyses primarily characterize repeatability within the particular bench setup and do not provide device-level population inference regarding 14-G catheters or 8-Fr cannulas in general. I suggest reconsidering the emphasis placed on inferential hypothesis testing and more clearly emphasizing descriptive estimates and measurement variability. If the intent is to make inference at the device-type level, multiple independent physical specimens of each device would be required. |
| --- | --- |
| **Response** | We thank the reviewer for this important comment. We agree that the six observations per condition were technical replicates obtained using the same physical device and circuit setup and therefore do not provide independent device-level observations. Our intent was to characterize hydraulic contrasts and repeatability within the standardized bench setup rather than to make population-level inference regarding 14-G catheters or 8-Fr cannulas in general.  In response, we retained the statistical analyses but substantially reframed their role and interpretation. Throughout the revised manuscript, ANOVA, Welch’s *t*-tests, confidence intervals, and standardized effect sizes are now explicitly treated as exploratory summaries of the technical measurements obtained within the present bench setup. We placed greater emphasis on descriptive quantities, including means, standard deviations, and absolute pressure differences, and reduced the emphasis on hypothesis-test results and large standardized effect sizes in the narrative Results and Discussion. We also revised the terminology from “device types” to “tested device configurations” where appropriate and describe partial eta-squared as a descriptive measure of between-configuration separation within the present bench dataset.  We further strengthened the Limitations section to state explicitly that the present analyses do not support device-to-device, device-type population, or animal-level inference and that multiple independent physical specimens of each device type would be required for population-level inference regarding device performance. We believe these revisions more accurately align the statistical interpretation with the technical-replicate design of the study. |
| **Manuscript changes** | We revised the Statistical analysis, Results, Discussion, Limitations, and Supporting Information descriptions to emphasize descriptive findings and bench repeatability and to clarify that the inferential analyses are exploratory and do not support device-level population inference. We also standardized the wording in Table 2, Table 3, and the Fig 2 caption to identify *n* = 6 explicitly as technical replicates.  **Revised text in the Materials and Methods section, “Statistical analysis” subsection:**  “Because the six observations per condition were technical replicates obtained using the same physical device and circuit setup, all inferential analyses were treated as exploratory summaries of within-setup contrasts and repeatability rather than as tests supporting device-level population inference. Accordingly, interpretation emphasized means, SDs, and absolute differences, whereas confidence intervals, *p* values, and standardized effect sizes were treated as exploratory summaries of the technical measurements.”  **Revised description of the ANOVA and post-hoc analyses in the Materials and Methods section:**  “Exploratory one-way analysis of variance (ANOVA) was performed for each pump flow-rate setting (50–350 mL/min) to compare outlet pressure among the four configurations included in the primary device comparison: the 8-Fr cannula and the side-hole 14-G 25, 14-G 32, and 14-G 38 specimens. When the ANOVA outcome was significant (*p* ≤ 0.05), exploratory post-hoc pairwise comparisons versus the 8-Fr cannula were conducted using Welch’s two-sample *t*-test. Partial eta-squared (*η*_p_^2^) is reported as a descriptive standardized measure of between-configuration separation within the present bench dataset. For families of pairwise Welch’s *t*-tests, *p* values were adjusted for multiple comparisons using the Holm method. Families were defined as: (i) device-versus-8-Fr comparisons at the 350 mL/min pump flow-rate setting (three tests), (ii) side-hole comparisons at the 300 mL/min pump flow-rate setting across catheter lengths (three tests), and (iii) exploratory side-hole comparisons across pump flow-rate settings within each catheter length (seven tests per length).”  **Revised significance-threshold statement in the Materials and Methods section:**  “All tests were two-tailed, and *p* ≤ 0.05 was used as the nominal significance threshold for the exploratory analyses.”  **Revised text in the Results section, “Outlet pressure across pump flow-rate settings” subsection:**  “Fig 2 shows the relationship between pump flow-rate setting and outlet pressure for the 8-Fr arterial cannula and the three side-hole 14-G hemodialysis catheter specimens (14-G 25, 14-G 32, and 14-G 38) in the mock 3-kg small-dog CPB circuit. Across the predefined primary analysis range of 50–300 mL/min, mean outlet pressures increased in a near-linear manner as the pump flow-rate setting increased and remained closely clustered among the four tested configurations at each pump flow-rate setting (Fig 2). Exploratory ANOVA performed separately at each pump flow-rate setting did not identify statistically detectable between-configuration separation over this range (*F*(3, 20) ≤ 1.52, *p* ≥ 0.241, partial *η*_p_^2^ ≤ 0.19). At the exploratory 350 mL/min pump flow-rate setting, mean outlet pressures separated more clearly among configurations, and exploratory ANOVA yielded *F*(3, 20) = 164.87, *p* < 0.001, and partial *η*_p_^2^ = 0.96. Pairwise contrasts versus the 8-Fr cannula at the 350 mL/min pump flow-rate setting are provided in S2 Table. These inferential statistics are presented as exploratory summaries of the technical measurements obtained within the present bench setup. Detailed ANOVA outputs and pairwise estimates are provided in S1 and S2 Tables, respectively.”  **Revised text in the Discussion section, “Summary of main findings” subsection:**  “Across the 50–300 mL/min pump flow-rate setting range, mean outlet pressures remained closely clustered among the tested configurations, and exploratory ANOVA performed separately at each pump flow-rate setting did not identify statistically detectable between-configuration separation (S1 Table). At the exploratory 350 mL/min pump flow-rate setting, a clearer separation in mean outlet pressure was observed, with all three side-hole 14-G specimens showing higher values than the 8-Fr cannula (S2 Table). These statistical comparisons characterize the present technical measurements and should not be interpreted as device-level population inference.”  **Revised Third limitation in the Discussion section, “Limitations and future directions” subsection:**  “Third, six replicates per condition represented technical replicates obtained using the same physical device and circuit setup; manufacturing variability and between-animal anatomical variability were not assessed. Accordingly, the reported confidence intervals, *p* values, and standardized effect sizes should be interpreted as exploratory summaries of contrasts and repeatability within the present bench setup and not as device-to-device, device-type population, or animal-level inference. Multiple independent physical specimens of each device type would be required to support population-level inference regarding device performance. Future research should therefore include multiple samples per device type and consider formal equivalence testing where the goal is to conclude practical similarity within predefined margins [24].”  **Revised Supporting Information titles in the manuscript:**  “S1 Table. Exploratory one-way ANOVA of outlet pressure at each pump flow-rate setting among the 8-Fr cannula and three side-hole 14-G specimens in the mock 3-kg small-dog cardiopulmonary bypass circuit.”  “S2 Table. Exploratory post-hoc comparisons of outlet pressure at the 350 mL/min pump flow-rate setting between three side-hole 14-G specimens and the 8-Fr arterial cannula.” |

| **Reviewer comment** | 3. Absence of statistical significance should not be interpreted as evidence of equivalence or similarity. The manuscript appropriately states that formal equivalence was not established. However, several passages emphasize the absence of a statistically significant device effect and the fact that the 95% confidence intervals span zero. Given the technical-replicate design, and more generally because a non-significant result does not establish equivalence, I recommend that the authors place greater emphasis on the observed absolute pressure differences and their uncertainty rather than on non-significant *p* values. Statements such as “no device effect” should be carefully limited to the present experimental setup. |
| --- | --- |
| **Response** | We thank the reviewer for this important comment. We agree that the absence of statistical significance does not establish equivalence or similarity, particularly in the present study in which the observations were technical replicates obtained using the same physical devices and circuit setup. We therefore revised the manuscript to avoid interpreting non-significant hypothesis-test results or confidence intervals spanning zero as evidence of equivalence.  In the revised manuscript, we place greater emphasis on the observed absolute outlet-pressure differences and their uncertainty. At the 300 mL/min pump flow-rate setting, the mean outlet-pressure differences versus the 8-Fr cannula were +0.33 to +0.67 mmHg, with corresponding 95% confidence intervals ranging from −1.33 to +2.15 mmHg. We now explicitly state that no equivalence margin was prespecified and that these estimates should be interpreted as descriptive measures of the observed bench-level differences and uncertainty rather than as demonstrating formal equivalence.  We also revised the study hypothesis to avoid implying a formal test of practical equivalence, removed wording that could suggest that non-significant statistical findings established similarity, and rewrote the Conclusions to emphasize quantitative circuit-level observations rather than statistical non-significance. The revised Conclusions explicitly state that the findings do not establish device-level equivalence, interchangeability, or clinical validity, and emphasize the need for multiple independent physical specimens, clinically relevant validation models, and formal equivalence testing where predefined device-level similarity is the intended conclusion. |
| **Manuscript changes** | We revised the Abstract, Introduction, Results, Discussion, and Conclusions to emphasize the observed absolute pressure differences and their uncertainty and to avoid interpreting statistical non-significance as evidence of equivalence or similarity.  **Revised text in the Abstract:**  “Within the primary analysis range, outlet pressure was approximately 136–137 mmHg at 300 mL/min; mean differences for the 14-G specimens versus the 8-French cannula were +0.33 to +0.67 mmHg, with 95% confidence intervals from −1.33 to +2.15 mmHg.”  **Revised hypothesis statement in the Introduction:**  “In this study, we hypothesized that, within the prespecified primary analysis range of the present mock small-dog CPB circuit, the observed outlet-pressure differences between the three side-hole 14-G specimens and the 8-Fr pediatric arterial cannula would be small in magnitude.”  **Revised text in the Results section, “Primary comparison at the 300 mL/min pump flow-rate setting” subsection:**  “At the 300 mL/min pump flow-rate setting, mean outlet-pressure differences versus the 8-Fr cannula were +0.33 to +0.67 mmHg, with corresponding 95% CIs ranging from −1.33 to +2.15 mmHg. Because no equivalence margin was prespecified, these estimates are reported as descriptive measures of the observed differences and uncertainty within the present bench setup and should not be interpreted as demonstrating formal equivalence.”  **Revised text in the Results section, “Apparent hydraulic resistance and geometric indices” subsection:**  “Despite these theoretical differences in diameter-based indices, mean outlet-pressure differences for the three side-hole 14-G specimens versus the 8-Fr cannula at the 300 mL/min pump flow-rate setting were +0.33 to +0.67 mmHg, and *R*_app_ values were 0.25–0.26 mmHg/(mL/min) across these four tested configurations (Table 2; Figs 2 and 3).”  **Revised text in the Discussion section, “Summary of main findings” subsection:**  “At the prespecified reference pump flow-rate setting of 300 mL/min, mean outlet-pressure differences versus the 8-Fr cannula were +0.33 to +0.67 mmHg, with corresponding 95% CIs ranging from −1.33 to +2.15 mmHg (Table 2). These estimates describe the magnitude and uncertainty of the observed bench-level differences and do not establish formal equivalence.”  **Additional revised text in the Discussion section, “Summary of main findings” subsection:**  “Although the 14-G catheters have a substantially higher theoretical diameter-based resistance factor (Table 4), the observed mean outlet-pressure differences versus the 8-Fr cannula were ≤0.67 mmHg at the 300 mL/min pump flow-rate setting under the present bench conditions.”  **Revised Conclusions:**  “This in vitro bench study provides circuit-level hydraulic reference data for one 8-Fr pediatric arterial cannula and six 14-G hemodialysis catheter specimens at effective lengths of 25, 32, and 38 mm, with separate side-hole and sealed specimens at each length. Across the prespecified 50–300 mL/min pump flow-rate setting range, outlet-pressure differences between the three side-hole 14-G specimens and the 8-Fr cannula were small under the present bench conditions; at the 300 mL/min pump flow-rate setting, mean differences versus the 8-Fr cannula were +0.33 to +0.67 mmHg. Among the tested 14-G specimens, outlet pressure was 2.7–2.8 mmHg lower for the side-hole specimens than for the sealed specimens at 300 mL/min. At the exploratory 350 mL/min pump flow-rate setting, all three side-hole 14-G specimens showed higher outlet pressures than the 8-Fr cannula. These technical-replicate findings do not establish device-level equivalence, interchangeability, or clinical validity. Future studies should evaluate multiple independent physical specimens and clinically relevant validation models, with formal equivalence testing where predefined device-level similarity is the intended conclusion.” |

| **Reviewer comment** | 4. Statistical analysis of the side-hole experiment. The side-hole and sealed conditions appear to have been evaluated using the same physical catheter, whereas the analysis uses Welch's two-sample *t*-test. Please clarify whether measurements under the two configurations were paired or otherwise blocked by experimental run. If a natural pairing exists, a paired or repeated-measures analysis may be more appropriate. More importantly, because these remain technical measurements on the same physical catheter, the statistical inference should again be limited to bench repeatability. I would also recommend emphasizing the absolute difference of approximately 2–3 mmHg (approximately 2%) rather than the very large Hedges' *g* values, which appear to be driven largely by the very small within-condition variability of the bench experiment. |
| --- | --- |
| **Response** | We thank the reviewer for this important comment. We rechecked the experimental design and clarified that the side-hole and sealed conditions were not evaluated using the same physical catheter. For each effective length (25, 32, and 38 mm), one physical 14-G catheter was tested with the original side holes and a separate physical 14-G catheter was tested in the sealed condition, resulting in six physical 14-G catheters in total. The 8-Fr arterial cannula was tested first, followed by the 14-G catheters in order of effective length: 25, 32, and 38 mm. At each effective length, the side-hole specimen was tested before the separate specimen assigned to the sealed condition. Within each specimen/configuration, the pump flow-rate setting was increased sequentially from 50 to 350 mL/min in 50-mL/min increments, and this full sequence was repeated six times, yielding six technical replicates at each pump flow-rate setting. Because the two conditions at each effective length were evaluated using separate physical catheters, the measurements were not paired by physical specimen or experimental run, and there was no natural paired or repeated-measures structure. We therefore retained Welch’s two-sample *t*-test as an exploratory comparison of the technical measurements obtained from the two tested specimens at each effective length.  We agree that the six observations within each length-by-configuration condition were technical replicates and did not constitute six independent physical specimens. Because only one physical catheter represented each length-by-configuration condition, the observed differences between the side-hole and sealed specimens may reflect both configuration-related effects and specimen-specific variation and therefore should not be interpreted as an isolated causal effect of the side holes or as device-level population inference. We accordingly revised the manuscript to emphasize descriptive differences between the tested specimens and measurement variability within the present bench setup. We also shifted emphasis from standardized effect sizes to the absolute outlet-pressure difference: at the 300 mL/min pump flow-rate setting, outlet pressure was 2.7–2.8 mmHg lower for the tested side-hole specimens than for the sealed specimens, corresponding to an approximate difference of 2%. Hedges’ *g* is retained only as a descriptive standardized summary of the technical measurements and should not be interpreted as a device-level population effect. To further avoid overinterpretation of the large standardized effect-size values arising from the small within-condition variability, we removed the conventional small/medium/large interpretive thresholds from the Supporting Information. Because testing followed a fixed, non-randomized sequence, we also acknowledge that possible order-related or other time-dependent influences cannot be completely excluded. |
| **Manuscript changes** | We revised the Abstract, mock-circuit description, experimental procedure, definition of the secondary outcome, statistical analysis, Results, Table 3, Discussion, Limitations, and Supporting Information to clarify the physical-specimen structure and measurement sequence, emphasize the observed absolute differences, and narrow the scope of inference.  **Revised text in the Abstract:**  “This in vitro study evaluated one 8-French pediatric arterial cannula and six 14-gauge hemodialysis catheter specimens—separate side-hole and sealed specimens at effective lengths of 25, 32, and 38 mm—in a closed-loop mock circuit for a 3-kg small dog.”  “At 300 mL/min, outlet pressure was approximately 2–3 mmHg lower for the side-hole specimens than for the sealed specimens across all catheter lengths.”  **Revised text in the Materials and Methods section, “Mock small-dog cardiopulmonary bypass circuit” subsection:**  “One physical 8-Fr arterial cannula and six physical 14-G hemodialysis catheters were used. For each effective length (25, 32, and 38 mm), one catheter was tested with the original side holes and a separate catheter was tested in the sealed condition, resulting in six 14-G test specimens and seven physical test devices in total.”  **Revised text in the Materials and Methods section, “Experimental protocol and outcome measures” subsection:**  “For the 14-G catheters, two configurations were tested at each effective length (14-G 25, 14-G 32, and 14-G 38): (1) the original design with side holes and (2) a sealed condition in which the side-hole region was occluded by wrapping a short segment of non-elastic tape around the catheter. The 8-Fr arterial cannula was tested first, followed by the 14-G catheters in order of effective length: 25, 32, and 38 mm. At each effective length, the side-hole specimen was tested before the separate specimen assigned to the sealed condition. Within each configuration, the pump flow-rate setting was increased sequentially from 50 to 350 mL/min in 50-mL/min increments, and this full sequence was repeated six times, yielding six technical replicates at each pump flow-rate setting. Because the side-hole and sealed conditions at each effective length were evaluated using separate physical catheters, the measurements were not paired by physical specimen or experimental run. The configuration order was fixed rather than randomized or interleaved.”  **Revised text defining the secondary outcomes in the Materials and Methods section:**  “Secondary outcomes were outlet pressure across all pump flow-rate settings and outlet-pressure differences between the side-hole and sealed configurations at the 300 mL/min pump flow-rate setting.”  **Revised text in the Materials and Methods section, “Statistical analysis” subsection:**  “To compare the side-hole and sealed conditions, Welch’s two-sample *t*-test was used at each effective length because the two conditions were evaluated using separate physical catheters and the measurements were not paired by physical specimen or experimental run. Mean differences (side-hole value minus sealed value) and corresponding 95% confidence intervals (CIs) were estimated using Welch’s *t*-based method with Welch–Satterthwaite degrees of freedom. Hedges’ *g* was calculated as a descriptive standardized effect-size measure for each contrast. Because each length-by-configuration condition was represented by a single physical catheter and six technical replicates, these comparisons were intended to characterize differences between the tested specimens and measurement variability within the present bench setup and not to provide device-level population inference regarding the side-hole configuration.”  **Revised Results subsection heading:**  “Outlet-pressure differences between side-hole and sealed configurations at the 300 mL/min pump flow-rate setting”  **Revised text in the Results subsection:**  “Outlet-pressure differences between the side-hole and sealed configurations at the 300 mL/min pump flow-rate setting are summarized in Table 3. For all effective lengths, catheters with side holes showed lower outlet pressures than those without (sealed configuration) (14-G 25: 136.7 ± 1.37 vs 139.3 ± 1.21 mmHg; 14-G 32: 137.0 ± 1.10 vs 139.8 ± 1.17 mmHg; 14-G 38: 137.0 ± 0.89 vs 139.8 ± 0.75 mmHg). The respective mean differences (side-hole value minus sealed value) were −2.67 mmHg, −2.83 mmHg, and −2.83 mmHg, corresponding to an approximate difference of 2% relative to the sealed configuration. The corresponding 95% CIs and exploratory statistical comparisons are presented in Table 3. Because each length-by-configuration condition was represented by one physical catheter, the comparisons characterize differences between the tested specimens and measurement variability within the present bench setup rather than an isolated side-hole effect or a device-level population effect. A summary of side-hole differences across all tested pump flow-rate settings is provided in S3 Table.”  **Revised Table 3 title:**  “Table 3. Outlet pressure with side holes versus the sealed configuration at the 300 mL/min pump flow-rate setting in 14-G catheters.”  **Revised Table 3 note:**  “Hedges’ *g* is reported as the absolute value as a descriptive standardized summary of the technical measurements and should not be interpreted as a device-level population effect.”  **Revised text in the Discussion section, “Summary of main findings” subsection:**  “A modest but consistent difference between the side-hole and sealed configurations was observed at the 300 mL/min pump flow-rate setting: outlet pressure was 2.7–2.8 mmHg lower with side holes than with the sealed configuration (Table 3), corresponding to an approximate difference of 2%.”  **Revised Discussion subsection heading:**  “Outlet-pressure differences between side-hole configurations”  **Revised text in the Discussion section, “Outlet-pressure differences between side-hole configurations” subsection:**  “At the 300 mL/min pump flow-rate setting, outlet pressure was approximately 2.7–2.8 mmHg lower with side holes than with the sealed configuration across the three tested effective lengths (Table 3), corresponding to an absolute difference of approximately 2%. A possible explanation is redistribution of flow between the distal opening and side holes, which may alter local flow structures near the catheter tip [12,13]. However, local velocity fields, flow partitioning among individual openings, and near-tip pressure distributions were not measured directly in the present study; therefore, this explanation should be regarded as a mechanistic hypothesis rather than a directly demonstrated mechanism. Because the side-hole and sealed conditions at each effective length were tested using separate physical catheters, the observed differences may reflect both configuration-related effects and specimen-specific variation. Accordingly, these findings should be interpreted as descriptive contrasts between the tested specimens under the present bench conditions rather than as an isolated causal effect of the side holes or as a device-level population effect.”  **New Fourth limitation in the Discussion section, “Limitations and future directions” subsection:**  “Fourth, only one physical 14-G catheter was tested for each combination of effective length and side-hole configuration, and the side-hole and sealed conditions at a given effective length were evaluated using separate physical catheters. In addition, testing followed a fixed sequence: the 8-Fr cannula was tested first, followed by the 14-G catheters at effective lengths of 25, 32, and 38 mm, with the side-hole specimen tested before the sealed specimen at each length. The conditions were not randomized or interleaved. Therefore, the observed differences between side-hole configurations cannot be separated completely from specimen-specific variation or possible order-related or other time-dependent influences. These findings should not be interpreted as an isolated causal effect of the side holes. Future studies should include multiple independent physical specimens for each length and configuration and use randomized or counterbalanced experimental sequences where technically feasible.”  **Renumbering of the pre-existing limitation regarding the sealed configuration:**  Because the new specimen-structure and measurement-order limitation was inserted as the Fourth limitation, the previous Fourth limitation was retained and renumbered as Fifth. The revised text is: “Fifth, the sealed condition was created by external taping to occlude the side holes, which may have altered local stiffness or outer geometry. Purpose-built catheters without side holes, flow visualization, or both would better isolate the contribution of side-hole outflow [12,13].”  **Revised Supporting Information title:**  “S3 Table. Outlet-pressure differences between side-hole and sealed configurations across all pump flow-rate settings in 14-G hemodialysis catheters.”  **Revised Supporting Information notes:**  In both the S2 and S3 Table notes, Hedges’ *g* is described as the absolute value and as a descriptive standardized summary of the technical measurements that should not be interpreted as a device-level population effect. The conventional small/medium/large interpretive thresholds were removed to avoid overemphasizing standardized effect-size magnitudes derived from the technical-replicate measurements. The S1–S3 notes were also clarified to state explicitly that the reported observation counts represent technical measurements or technical replicates rather than independent physical devices. |

| **Reviewer comment** | 5. Clinical interpretation of the 300 mL/min reference flow and the term “supraphysiologic.” The manuscript derives the 300 mL/min reference flow from a perfusion index of 1.4 L/min/m². Please provide slightly more clinical context for this choice, including how representative this flow is of current CPB practice in approximately 3-kg dogs under the stated hypothermic conditions. Unless there is strong clinical evidence that 350 mL/min is physiologically excessive in this setting, “above the prespecified flow range” or similar wording may be preferable to the broader term “supraphysiologic.” |
| --- | --- |
| **Response** | We thank the reviewer for this important comment. We agree that the 300 mL/min reference pump flow-rate setting should not be presented as a universal physiologic target and that the term “supraphysiologic” was too strong for the exploratory 350 mL/min pump flow-rate setting without direct evidence that this setting represents a physiologically excessive flow in approximately 3-kg dogs.  In response, we added clinical context regarding reported CPB flows in small and toy-breed dogs. In a previously reported 2.4-kg toy-breed dog managed under moderate hypothermia (minimum rectal temperature, 26.6°C; target minimum, 28°C), pump flow was 280 mL/min before aortic cross-clamping and 150–230 mL/min during cross-clamping, with a target maximum flow of 370 mL/min. We therefore clarify that the 300 mL/min reference pump flow-rate setting used in the present 3-kg bench model is of the same order as a clinically reported flow in a similarly sized dog, but should not be interpreted as a universal target across CPB phases. We also cite an earlier series of small and toy-breed dogs undergoing deeper hypothermia, in which the mean lowest pump flow during aortic cross-clamping was 29.2 mL/kg/min, further illustrating that CPB flow requirements vary with temperature and operative phase.  Accordingly, we now describe the perfusion index of 1.4 L/min/m² as a pragmatic low-flow reference for the present bench model rather than as a universal physiologic or clinical target. We also replaced the terminology “physiologic range” with “prespecified primary analysis range” where appropriate and removed the term “supraphysiologic.” The 350 mL/min pump flow-rate setting is now described as an exploratory setting above the prespecified primary analysis range. |
| **Manuscript changes** | We revised the Abstract, Introduction, Materials and Methods, Fig 2 caption, and Discussion to provide additional clinical context for the 300 mL/min reference pump flow-rate setting, to clarify that this value is a pragmatic study reference rather than a universal physiologic target, and to replace “supraphysiologic” terminology with wording that reflects the prespecified study range.  **Revised text in the Abstract:**  “The Stockert S5 pump flow-rate setting was increased from 50 to 350 mL/min; 50–300 mL/min was prespecified as the primary analysis range and 350 mL/min as exploratory.”  “Within the primary analysis range, outlet pressure was approximately 136–137 mmHg at 300 mL/min; mean differences for the 14-G specimens versus the 8-French cannula were +0.33 to +0.67 mmHg, with 95% confidence intervals from −1.33 to +2.15 mmHg.”  **Revised hypothesis statement in the Introduction:**  “In this study, we hypothesized that, within the prespecified primary analysis range of the present mock small-dog CPB circuit, the observed outlet-pressure differences between the three side-hole 14-G specimens and the 8-Fr pediatric arterial cannula would be small in magnitude.”  **Revised text in the Materials and Methods section, “Circuit scaling and target flow for the 3-kg small-dog model” subsection:**  “Veterinary CPB flow requirements vary with body size, temperature, hemodilution, and phase of bypass [4,5]. In a reported 2.4-kg toy-breed dog managed under moderate hypothermia (minimum rectal temperature, 26.6°C; target minimum, 28°C), pump flow was 280 mL/min before aortic cross-clamping and 150–230 mL/min during cross-clamping; the target maximum flow was 370 mL/min [11]. Thus, the 300 mL/min reference used in the present bench model is of the same order as the approximately 280 mL/min flow reported before aortic cross-clamping in a similarly sized 2.4-kg dog under moderate hypothermia, but it should not be interpreted as a universal target across CPB phases. In an earlier series of small and toy-breed dogs undergoing deeper hypothermia (mean lowest esophageal temperature, 21.4°C), the mean lowest pump flow during aortic cross-clamping was 29.2 mL/kg/min [5]. These reports indicate that reported perfusion flows vary substantially with temperature and operative phase and do not define a single universal physiologic upper limit. Accordingly, *PI*_eff_ was prespecified at 1.4 L/min/m^2^ as a pragmatic low-flow reference for the present 3-kg bench model rather than as a universal physiologic or clinical target. For a 3-kg model (BSA = 0.21 m^2^), Equation 2 yielded *Q*_ref_ = 294 mL/min, which was rounded to 300 mL/min as the reference pump flow-rate setting for subsequent testing.”  **Revised text in the Fig 2 caption:**  “The vertical dotted line indicates 300 mL/min (upper limit of the predefined primary analysis range); 350 mL/min was treated as an exploratory condition above the prespecified primary analysis range.”  **Revised text in the Discussion section, “Summary of main findings” subsection:**  “At the prespecified reference pump flow-rate setting of 300 mL/min, mean outlet-pressure differences versus the 8-Fr cannula were +0.33 to +0.67 mmHg, with corresponding 95% CIs ranging from −1.33 to +2.15 mmHg (Table 2). These estimates describe the magnitude and uncertainty of the observed bench-level differences and do not establish formal equivalence.”  **Revised text in the Discussion section, “Relevance to small-dog cardiopulmonary bypass” subsection:**  “Within the prespecified 50–300 mL/min pump flow-rate setting range, the three side-hole 14-G specimens produced outlet pressure and *R*_app_ values with only small observed differences compared with those of the 8-Fr cannula in this mock circuit. However, because the present experiments used technical replicates in a bench model, these data should be interpreted as circuit-level hydraulic benchmarks within the standardized circuit and not as establishing clinical interchangeability or device-to-device equivalence [4,5,11].” |

| **Reviewer comment** | 6. Comparison with hemodialysis pressure values should be reconsidered. The Discussion compares the approximately 135-mmHg pressure observed in the mock CPB circuit with pressure values reported during hemodialysis vascular-access surveillance. The circuit configuration, pressure measurement location, access conditions, and clinical significance of these measurements differ considerably between hemodialysis and CPB. I am concerned that this numerical comparison could unintentionally imply that the observed pressure is clinically acceptable or safe. I suggest removing this comparison or substantially de-emphasizing it unless a stronger physiological rationale can be provided. |
| --- | --- |
| **Response** | We thank the reviewer for this important comment. We agree that direct numerical comparison between the outlet pressures measured in the present mock CPB circuit and pressures reported during hemodialysis vascular-access surveillance could be misleading because the circuit configuration, pressure-measurement location, access conditions, and clinical interpretation differ substantially between the two settings. We also agree that such a comparison could unintentionally imply that the approximately 135-mmHg outlet pressure observed in the present bench model represents a clinically acceptable or safe pressure level.  In response, we removed the hemodialysis pressure comparison from the Discussion and deleted the two references that were used solely to support that comparison. We instead added an explicit statement that the absolute outlet-pressure values observed in this study depend on the specific mock-circuit configuration and pressure-measurement location and should be interpreted only as standardized circuit-level hydraulic measurements, not as evidence of clinically acceptable or safe pressure levels. The reference list and corresponding in-text citations were updated accordingly. |
| **Manuscript changes** | We removed the numerical comparison between the outlet pressures measured in the present mock CPB circuit and pressures reported during hemodialysis vascular-access surveillance. We also added an explicit statement limiting interpretation of the absolute pressure values to the present standardized bench configuration and clarified that they should not be interpreted as evidence of clinical safety or acceptability.  **Deleted text from the Discussion section, “Relevance to small-dog cardiopulmonary bypass” subsection:**  “Typical pressures reported during vascular access surveillance in hemodialysis can exceed 120–150 mmHg at blood flows of 200–400 mL/min [18,19], whereas outlet pressure in the present mock CPB circuit was approximately 135 mmHg at 300 mL/min. Although direct extrapolation from hemodialysis to CPB was limited, the pressure range observed here provides contextual support that the tested 14-G catheters can operate within a measurable low-flow hydraulic range under the present standardized bench conditions.”  **Added text in the Discussion section, “Relevance to small-dog cardiopulmonary bypass” subsection:**  “Because the absolute outlet-pressure values in this study depend on the specific mock-circuit configuration and pressure-measurement location, they should be interpreted only as standardized circuit-level hydraulic measurements and not as evidence of clinically acceptable or safe pressure levels.”  **Reference-list and in-text citation changes:**  The two references used only for the hemodialysis pressure comparison were removed:  “Smits JH, van der Linden J, Hagen EC, Modderkolk-Cammeraat EC, Feith GW, Koomans HA, et al. Graft surveillance: venous pressure, access flow, or the combination? Kidney Int. 2001;59(4): 1551–1558.”  “Hoeben H, Abu-Alfa AK, Reilly RF, Aruny JE, Bouman K, Perazella MA. Vascular access surveillance: evaluation of combining dynamic venous pressure and vascular access blood flow measurements. Am J Nephrol. 2003;23(6): 403–408.”  The reference list and corresponding in-text citations were updated consistently throughout the final revised manuscript. Because two manufacturer documents were subsequently added as References 16 and 17 in response to Reviewer #2, Major Comment 1, and the fixed flow rate–injection pressure method reference was subsequently added as Reference 20, the references that were previously numbered 20–24 were renumbered as References 21–25 in the final revised manuscript. |

**Minor Revision**

| **Reviewer comment** | 7. Inconsistent *p* values for the side-hole comparisons. The Holm-adjusted *p* values reported in the Results text are 0.005, 0.003, and <0.001, whereas Table 3 reports 0.010, 0.006, and <0.001. Please verify the analysis and correct the inconsistent values. |
| --- | --- |
| **Response** | We thank the reviewer for identifying this inconsistency. We rechecked the analysis and confirmed that, for the side-hole comparisons at the 300 mL/min pump flow-rate setting, the Holm adjustment should be applied across the three prespecified length-specific comparisons. The correct Holm-adjusted *p* values are 0.005 for 14-G 25, 0.003 for 14-G 32, and < 0.001 for 14-G 38. We corrected Table 3 accordingly. We also verified that the statistical-analysis description now explicitly defines this family as the three side-hole comparisons at the 300 mL/min pump flow-rate setting across catheter lengths.  The *p* values in S3 Table were not changed because that table reports a separate exploratory comparison family in which Holm adjustment was applied across the seven pump flow-rate settings within each catheter length. |
| **Manuscript changes** | We corrected the Holm-adjusted *p* values in Table 3 to match the prespecified three-comparison family at the 300 mL/min pump flow-rate setting.  Corrected values in Table 3:   - 14-G 25: 0.010 → 0.005 - 14-G 32: 0.006 → 0.003 - 14-G 38: < 0.001 (unchanged)   The corresponding Table 3 note states:  “Holm-adjusted *p*-values were obtained using Welch’s two-sample *t*-test comparing catheters with side holes and those without (sealed configuration) at the 300 mL/min pump flow-rate setting, with adjustment across the three prespecified length-specific comparisons.”  The Statistical analysis subsection likewise defines this comparison family as:  “(ii) side-hole comparisons at the 300 mL/min pump flow-rate setting across catheter lengths (three tests)”  No changes were made to the corresponding *p* values in S3 Table because they were calculated using the separate seven-comparison family across pump flow-rate settings within each catheter length. |

| **Reviewer comment** | 8. Figure 1 would benefit from a clear flow-direction arrow and clearer labeling of the pressure measurement sites. From a clinical reader's perspective, it would be helpful to indicate explicitly which pressure corresponds to the regulated 60-mmHg site, which represents the reported outlet pressure, and the direction of perfusate flow. This would make the experimental setup considerably easier to understand. |
| --- | --- |
| **Response** | We thank the reviewer for this helpful suggestion. We revised Figure 1 to make the mock-circuit configuration easier to interpret clinically. Specifically, we added arrows indicating the direction of perfusate flow and clearly labeled the two pressure-measurement sites as the regulated inlet pressure (*P*_in_) and the reported outlet pressure (*P*_out_). We also indicated the respective distances of these sites from the test device.  During this revision, we also rechecked the orientation of the pressure-measurement sites relative to the actual direction of perfusate flow. To ensure consistency between the figure, the manuscript, and the retained trial-level dataset, we clarified throughout the revised text that *P*_out_ was recorded 1,000 mm upstream of the test device, whereas *P*_in_ was monitored 300 mm downstream and regulated to 60 mmHg. We further clarified that trial-level *P*_in_ values were not retained and that Δ*P*_seg_ and *R*_app_ were calculated using the regulated *P*_in_ target of 60 mmHg. We also revised descriptions of the measured circuit segment accordingly. |
| **Manuscript changes** | We revised Figure 1 and the corresponding manuscript text to clarify the direction of perfusate flow, the locations of the pressure-measurement sites, and the distinction between the regulated inlet pressure and the reported outlet pressure.  **Figure 1 changes:**   - Added arrows indicating the direction of perfusate flow. - Labeled the regulated pressure site as:   “Inlet pressure (*P*_in_): regulated to 60 mmHg”   - Labeled the reported pressure site as:   “Outlet pressure (*P*_out_): reported outlet pressure”   - Indicated the measurement-site distances as:   “300 mm downstream of test device”  and  “1,000 mm upstream of test device”   - Revised the device label to:   “Test device (14-G or 8-Fr)”  **Revised text in the Abstract:**  “The reported outlet pressure was recorded 1,000 mm upstream of the test device, while inlet pressure was monitored 300 mm downstream and regulated to 60 mmHg; six technical runs per condition were conducted using the same physical device and circuit setup.”  **Revised text in the Introduction:**  “Moreover, idealized tube-flow calculations do not fully capture device performance in practice. Real devices incorporate local geometric features, such as tapering tips and side holes, which introduce localized losses and flow redistribution; accordingly, outlet pressure within a standardized circuit segment cannot be inferred only from idealized calculations [3].”  “Therefore, this in vitro study aimed to quantify outlet pressure characteristics of an 8-French (Fr) pediatric arterial cannula and 14-G hemodialysis catheters within a standardized measured circuit segment of a closed-loop mock CPB circuit scaled to reproduce hemodynamic conditions expected for a 3-kg small dog. Specifically, we evaluated the combined hydraulic behavior of each device and the tubing between the two pressure-measurement sites under low-flow conditions representative of small-dog CPB, while systematically varying the pump flow-rate setting, effective length, and side-hole configuration.”  **Revised text in the Materials and Methods section, “Mock small-dog cardiopulmonary bypass circuit” subsection:**  “Inlet pressure (*P*_in_) was monitored 300 mm downstream of the test device and regulated to 60 mmHg, whereas the reported outlet pressure (*P*_out_) was recorded 1,000 mm upstream of the test device. In this study, *P*_in_ and *P*_out_ denote the designated circuit pressure-monitoring sites and do not refer to the inlet and outlet of the test device. A pressure-regulating tubing clamp was located downstream of the *P*_in_ measurement site, whereas a separate circuit-closure clamp was used only to maintain the closed-loop configuration and prevent loss of perfusate. These measurement sites are labeled in Fig 1, and arrows indicate the direction of perfusate flow. This configuration was intended to represent arterial-line pressure monitoring in small-dog CPB [4–6]. Accordingly, the calculated pressure difference between the recorded *P*_out_ value and the regulated *P*_in_ target reflects the combined hydraulic behavior of the test device and the tubing between the two pressure-measurement sites rather than device-intrinsic resistance alone.”  **Revised Figure 1 caption:**  “Fig 1. Mock small-dog cardiopulmonary bypass circuit schematic. The closed-loop circuit comprises a roller pump, polyvinyl chloride tubing with an inner diameter of 1/4 inch, tube clamps, two pressure transducers and two physiologic monitors, a temperature sensor and temperature control unit, and the test arterial access device (14-G hemodialysis catheter or 8-Fr pediatric arterial cannula). Each test device was inserted into the arterial-line tubing such that approximately 10 mm of its distal portion extended into the tubing lumen. Arrows indicate the direction of perfusate flow. Inlet pressure (*P*_in_), monitored 300 mm downstream of the test device, was regulated to 60 mmHg before each recording by manual adjustment of the pressure-regulating tubing clamp. The separate circuit-closure clamp was used only to maintain the closed-loop configuration and prevent loss of perfusate. Outlet pressure (*P*_out_), recorded 1,000 mm upstream of the test device, was the pressure outcome reported in this study.”  **Revised text in the Materials and Methods section, “Theoretical geometric indices” subsection:**  “Considering the tested cannulas/catheters include localized losses (e.g., entrance/exit and side-hole effects) and the calculated pressure difference reflects the test device plus the tubing between the two pressure-measurement sites, Equation (3) was not used to predict outlet pressure directly.”  **Revised definition of the calculated pressure difference in the Materials and Methods section:**  “where *P*_out_ denotes the recorded pressure measured 1,000 mm upstream of the test device and *P*_in_ denotes the regulated target pressure of 60 mmHg monitored 300 mm downstream of the test device. Trial-level *P*_in_ values were not retained; therefore, Δ*P*_seg_ was calculated using the regulated *P*_in_ target of 60 mmHg.”  **Revised text in the Results section, “Primary comparison at the 300 mL/min pump flow-rate setting” subsection:**  “The corresponding calculated Δ*P*_seg_, using the regulated *P*_in_ target of 60 mmHg, ranged 76.3–77.0 mmHg, and *R*_app_ was 0.25–0.26 mmHg/(mL/min) for all four primary-comparison configurations.”  **Revised Table 2 note:**  “Δ*P*_seg_ was calculated as *P*_out_ − 60 mmHg, where *P*_out_ was recorded 1,000 mm upstream of the test device and 60 mmHg was the regulated *P*_in_ target monitored 300 mm downstream of the test device. Trial-level *P*_in_ values were not retained. *R*_app_ was defined as Δ*P*_seg_ divided by the pump flow-rate setting displayed on the Stockert S5 and expressed in mmHg/(mL/min); it was therefore interpreted as a setting-based apparent hydraulic index rather than as resistance calculated from independently measured circuit flow.”  **Revised text in the Discussion section, “Hydraulic determinants and comparison with theory” subsection:**  “The calculated pressure difference, Δ*P*_seg_, reflects the combined hydraulic behavior of the test device and the tubing between the two pressure-measurement sites (1,000 mm upstream and 300 mm downstream of the test device), and the tested effective lengths (25–38 mm) are short relative to this measured circuit segment.”  **Revised Second limitation in the Discussion section:**  “Second, the mock circuit lacked vascular compliance, pulsatility, and autoregulation, and pressures were measured 300 mm downstream and 1,000 mm upstream rather than immediately adjacent to the test device. This configuration was chosen to represent arterial-line pressure monitoring in the mock CPB circuit and to capture the combined hydraulic behavior of the test device and the tubing between the two pressure-measurement sites [4–6,9]. Consequently, the absolute values should be interpreted as standardized comparisons within the measured circuit segment rather than as direct surrogates for in vivo arterial pressure at the device tip. In addition, although *P*_in_ was adjusted to and confirmed at the 60 mmHg target before *P*_out_ recording, trial-level *P*_in_ values were not retained. Accordingly, Δ*P*_seg_ and *R*_app_ were calculated using the regulated *P*_in_ target of 60 mmHg rather than trial-level recorded *P*_in_ values. Actual circuit flow was not independently measured with a separate flow meter; experimental flow conditions were defined by the pump flow-rate settings displayed on the Stockert S5. Therefore, analyses involving flow, including *R*_app_, should be interpreted as setting-based bench comparisons rather than measurements based on independently verified circuit flow.” |

| **Reviewer comment** | 9. The statement that “some centers adopt 14-G hemodialysis catheters” may be somewhat stronger than the cited evidence supports. The manuscript appears to cite a limited clinical report of this off-label strategy. Wording such as “off-label use has been reported” may more accurately reflect the available evidence unless broader clinical use can be documented. |
| --- | --- |
| **Response** | We thank the reviewer for this helpful comment. We agree that the phrase “some centers adopt” could imply broader clinical use than is supported by the cited evidence. We therefore revised the Introduction to state more precisely that off-label use of 14-G hemodialysis catheters as alternative arterial access devices has been reported, without implying widespread adoption. We also streamlined the following sentence to avoid redundant wording while retaining the stated gap in quantitative hydraulic characterization. |
| **Manuscript changes** | We revised the corresponding wording in the Introduction to avoid implying broader clinical adoption than is supported by the cited evidence.  **Revised text in the Introduction:**  “Off-label use of 14-gauge (G) hemodialysis catheters as alternative arterial access devices in low-flow extracorporeal circuits has been reported [11]. However, quantitative characterization of pressure–flow behavior and side-hole effects under controlled CPB-relevant conditions remains limited, despite related computational analyses such as computational fluid dynamic simulations of dialysis catheters and cannula–vessel configurations [12,13].” |

| **Reviewer comment** | 10. The physiological justification for the chosen blood-analogue viscosity could be refined. The working-fluid viscosity was appropriately measured at 28°C and showed good repeatability. However, Reference 14 appears primarily to support the relationship between hemodilution and whole-blood viscosity rather than directly representing hypothermic, hemodiluted canine blood under CPB conditions. A more directly relevant reference would strengthen the physiological justification, or the authors could clarify that 2.8 cP was selected as an approximate rather than species- and temperature-specific blood analogue. |
| --- | --- |
| **Response** | We thank the reviewer for this helpful comment. We agree that Reference 14 does not specifically establish that a viscosity of 2.8 cP reproduces hypothermic, hemodiluted canine blood under CPB conditions. We therefore revised the manuscript to avoid implying species- and temperature-specific physiological equivalence. The working fluid is now described as an approximate Newtonian blood analogue used for standardized hydraulic testing, and we explicitly state that it was not intended to reproduce the species- and temperature-specific rheology of hypothermic, hemodiluted canine blood. We also revised the Abstract to describe the glycerol–water solution as an approximate Newtonian blood analogue. |
| **Manuscript changes** | We revised the Abstract and the Materials and Methods section to clarify that the glycerol–water solution was an approximate Newtonian blood analogue for standardized hydraulic testing rather than a species- and temperature-specific representation of hypothermic, hemodiluted canine blood.  **Revised text in the Abstract:**  “A 34% (w/w) glycerol–water solution (viscosity 2.8 cP at 28°C) served as an approximate Newtonian blood analogue.”  **Revised text in the Materials and Methods section, “Mock small-dog cardiopulmonary bypass circuit” subsection:**  “Because blood viscosity varies with hematocrit and exhibits non-Newtonian behavior [14,15], the working fluid was used only as an approximate Newtonian blood analogue for standardized hydraulic testing and was not intended to reproduce the species- and temperature-specific rheology of hypothermic, hemodiluted canine blood. These conditions minimized cellular and non-Newtonian effects, facilitating evaluation of geometric and hydraulic contributions to pressure loss.” |

| **Reviewer comment** | 11. Editorial correction. In the first paragraph of the Limitations section, “Therefore” is repeated in consecutive sentences. Please revise for readability. |
| --- | --- |
| **Response** | We thank the reviewer for identifying this editorial issue. We revised the first paragraph of the Limitations section to avoid repeating “Therefore” in consecutive sentences. We also corrected an additional redundant transitional phrase in the Discussion (“Thus ... therefore”) to improve readability and consistency. |
| **Manuscript changes** | We revised two transitional expressions in the Discussion for readability.  **Revised text in the Discussion section, “Hydraulic determinants and comparison with theory” subsection:**  “Thus, these results should be interpreted as circuit-level hydraulic comparisons rather than intrinsic measurements of device resistance alone.”  **Revised text in the Discussion section, “Limitations and future directions” subsection:**  “First, the experiments were performed using a Newtonian glycerol–water solution to isolate geometric and hydraulic effects [14,22]. Therefore, the non-Newtonian rheology of blood, its cellular components, and hemolysis were not evaluated. Accordingly, any statements regarding blood damage are limited to the hydraulic context only and do not constitute evidence of hemocompatibility; dedicated ex vivo or in vivo studies using blood are required to establish this point [1,2,23].” |

# Response to Reviewer #2

**Overall response to Reviewer #2**

We thank Reviewer #2 for the constructive comments and for considering the manuscript technically sound, the statistical analysis appropriate, the underlying data fully available, and the English clear. The reviewer requested additional information on device flow specifications, clarification of the relationship between clamp position and inlet pressure and the overall mock-circuit configuration, confirmation of device position, and a more streamlined Conclusion with clear directions for future research. Each point is addressed below.

**Major Comments**

| **Reviewer comment** | 1. What is the maximum blood flow rate that the arterial cannula and the hemodialysis catheters can be provided? It is best to indicate it in table 1. |
| --- | --- |
| **Response** | We thank the reviewer for this important comment. We rechecked the available manufacturer documentation for flow specifications of the tested devices. The documentation reviewed did not specify a maximum recommended blood-flow rate for either the 8-Fr pediatric arterial cannula or the 14-G hemodialysis catheter. For the pediatric one-piece arterial cannula, Medtronic provides pressure-loss curves as a function of water flow rather than a single maximum-flow rating. For the 14-G SafeTouch Cannula GA, Nipro reports an average flow of 343 mL/min based on a 38-mm effective length; however, this value is explicitly an average flow specification and should not be interpreted as a maximum recommended flow rate.  To avoid assigning unsupported maximum-flow values, we therefore added the available manufacturer-reported flow information to Table 1 and explicitly distinguished the reported average flow from a maximum-flow limit. We also clarified this distinction in the Materials and Methods section and added the corresponding manufacturer documents as References 16 and 17.  Importantly, these manufacturer-reported data were obtained under conditions different from those of the present study and do not provide a head-to-head comparison of the two devices in a standardized small-dog CPB circuit. The present study therefore addresses a distinct question by characterizing circuit-level pressure behavior across controlled pump flow-rate settings and by evaluating catheter-length and side-hole effects. |
| **Manuscript changes** | We revised the Materials and Methods section and Table 1 to report the available manufacturer flow specifications while clarifying that the manufacturer documentation reviewed did not provide a maximum recommended blood-flow rate for the tested devices.  **Added text in the Materials and Methods section, “Mock small-dog cardiopulmonary bypass circuit” subsection:**  “Manufacturer documentation was also reviewed for device flow specifications. The documentation reviewed did not specify a maximum recommended blood-flow rate for either tested device. Medtronic provides pressure-loss curves as a function of water flow for the pediatric one-piece arterial cannulae, whereas Nipro reports an average flow of 343 mL/min for the 14-G SafeTouch Cannula GA measured at an effective length of 38 mm; this value represents an average flow specification rather than a maximum-flow limit [16,17].”  **Revised Table 1 title:**  “Table 1. Geometric characteristics and manufacturer-reported flow information for the arterial cannula and 14-G hemodialysis catheters.”  **A new column, “Manufacturer-reported flow information,” was added to Table 1.** The entries are “Maximum not specified; pressure-loss curve provided” for the 8-Fr cannula, “Maximum not specified” for the 14-G 25 and 14-G 32 catheters, and “Average flow, 343 mL/min; maximum not specified” for the 14-G 38 catheter.  **The Table 1 note was expanded as follows:**  “Manufacturer documentation did not specify a maximum recommended blood-flow rate for either tested device. For the pediatric one-piece arterial cannula, Medtronic provides pressure-loss curves as a function of water flow rather than a maximum recommended blood-flow rate [16]. For the 14-G SafeTouch Cannula GA, Nipro reports an average flow of 343 mL/min based on a 38-mm effective length; this value is not specified as a maximum recommended flow rate [17].”  **Reference-list changes:**  Two manufacturer documents were added as References 16 and 17 to support the manufacturer-reported flow information. Consequently, the former References 16 and 17 (Cowgill and Drabkin; Badeer) were renumbered as References 18 and 19, respectively. The fixed flow rate–injection pressure method reference (Okumura et al.) was subsequently added as Reference 20. Accordingly, the references that were previously numbered 20–24 were renumbered as References 21–25 in the final revised manuscript. |

| **Reviewer comment** | 2. What's the relationship of the clamp position and the inlet pressure? This manuscript described that the inlet pressure was regulated at 60 mmHg by partially occluding the arterial tubing with a clamp; the clamp position was manually adjusted while monitoring the pressure display to maintain the target inlet pressure before each recording. Please provide a detailed explanation of the Mock small-dog cardiopulmonary bypass circuit, and indicate the direction of fluid flow in Figure 1. |
| --- | --- |
| **Response** | We thank the reviewer for this important comment. We agree that the relationship between clamp position and inlet pressure, as well as the distinct roles of the two clamps and the pressure-measurement procedure, required clearer explanation. The pressure-regulating tubing clamp was located downstream of the *P*_in_ measurement site. Partial occlusion of this tubing increased downstream hydraulic resistance and thereby increased *P*_in_, whereas reducing the degree of occlusion decreased *P*_in_. Two pressure transducers and two physiologic monitors were used, with one transducer–monitor pair for *P*_in_ and the other for *P*_out_, and both pressure transducers were zeroed to atmospheric pressure before testing. At each pump flow-rate setting, *P*_in_ was adjusted to the target of 60 mmHg while monitoring the pressure display. After *P*_in_ had been adjusted, the circuit was allowed to stabilize for at least 60 s; the 60 mmHg *P*_in_ target was then confirmed, and a single *P*_out_ value was recorded from the physiologic monitor. Trial-level *P*_in_ values were not retained; therefore, Δ*P*_seg_ and *R*_app_ were calculated using the regulated *P*_in_ target of 60 mmHg.  A separate circuit-closure clamp served a different purpose: it was used only to maintain the closed-loop configuration and prevent loss of the glycerol–water perfusate and was not used to regulate *P*_in_.  We also clarified that *P*_in_ and *P*_out_ are designated circuit pressure-monitoring labels and do not denote the inlet and outlet of the test device. We therefore revised the Materials and Methods section to clarify the locations and distinct functions of the two clamps, the use and zeroing of the two pressure transducers, the sequence of *P*_in_ regulation, stabilization, and *P*_out_ recording, and the basis for calculating Δ*P*_seg_ and *R*_app_. Figure 1 was also revised to distinguish the pressure-regulating tubing clamp from the circuit-closure clamp and to show the direction of perfusate flow and the *P*_in_ and *P*_out_ sites. |
| **Manuscript changes** | We revised the Materials and Methods section and Figure 1 to clarify the relationship between clamp position and *P*_in_, the distinct functions of the two clamps, the use and atmospheric zeroing of two pressure transducers, the sequence of *P*_in_ regulation, stabilization, and *P*_out_ recording, the non-retention of trial-level *P*_in_ values, and the direction of perfusate flow through the mock circuit. Revised equipment description in the Materials and Methods section, “Mock small-dog cardiopulmonary bypass circuit” subsection:  “The system consisted of a CPB machine with a roller pump and temperature sensor (Stockert S5; LivaNova Japan KK, Tokyo, Japan), two pressure transducers (TruWave; Edwards Lifesciences, Tokyo, Japan), two physiologic monitors (BSM-2300; Nihon Kohden Corporation, Tokyo, Japan), and a temperature control unit (HHC-51; Senko Medical Instrument Mfg. Co., Ltd., Tokyo, Japan).”  **Added clarification in the Materials and Methods section, “Mock small-dog cardiopulmonary bypass circuit” subsection:**  “In this study, *P*_in_ and *P*_out_ denote the designated circuit pressure-monitoring sites and do not refer to the inlet and outlet of the test device.”  **Added text in the Materials and Methods section, “Mock small-dog cardiopulmonary bypass circuit” subsection:**  “A pressure-regulating tubing clamp was located downstream of the *P*_in_ measurement site, whereas a separate circuit-closure clamp was used only to maintain the closed-loop configuration and prevent loss of perfusate.”  **Revised text in the Materials and Methods section, “Experimental protocol and outcome measures” subsection:**  “Circuit-fluid temperature was monitored using the temperature sensor of the Stockert S5 and maintained at 28°C using the HHC-51 temperature control unit. Before testing, both pressure transducers were zeroed to atmospheric pressure. At each pump flow-rate setting, *P*_in_ was regulated to 60 mmHg by manually adjusting the degree of occlusion of the pressure-regulating tubing clamp while monitoring the *P*_in_ pressure display. Increasing clamp occlusion increased downstream hydraulic resistance and thereby increased *P*_in_, whereas reducing the occlusion decreased *P*_in_. After *P*_in_ had been adjusted to the target value, the circuit was allowed to stabilize for at least 60 s. The target *P*_in_ of 60 mmHg was confirmed, and a single *P*_out_ value was then recorded from the physiologic monitor connected to the *P*_out_ pressure transducer at 28°C.”  **Added clarification of the Δ*P*_seg_ calculation in the Materials and Methods section:**  “where *P*_out_ denotes the recorded pressure measured 1,000 mm upstream of the test device and *P*_in_ denotes the regulated target pressure of 60 mmHg monitored 300 mm downstream of the test device. Trial-level *P*_in_ values were not retained; therefore, Δ*P*_seg_ was calculated using the regulated *P*_in_ target of 60 mmHg.”  **Figure 1 changes:**  The clamp used to regulate *P*_in_ was labeled “Pressure-regulating tubing clamp,” and the separate clamp used to maintain the closed-loop circuit was labeled “Circuit-closure clamp.” Figure 1 also shows arrows indicating the direction of perfusate flow and identifies the *P*_in_ monitoring site and *P*_out_ recording site and their respective distances from the test device. The Figure 1 caption was revised to specify that two pressure transducers were used.  **Revised Figure 1 caption:**  “Fig 1. Mock small-dog cardiopulmonary bypass circuit schematic. The closed-loop circuit comprises a roller pump, polyvinyl chloride tubing with an inner diameter of 1/4 inch, tube clamps, two pressure transducers and two physiologic monitors, a temperature sensor and temperature control unit, and the test arterial access device (14-G hemodialysis catheter or 8-Fr pediatric arterial cannula). Each test device was inserted into the arterial-line tubing such that approximately 10 mm of its distal portion extended into the tubing lumen. Arrows indicate the direction of perfusate flow. Inlet pressure (*P*_in_), monitored 300 mm downstream of the test device, was regulated to 60 mmHg before each recording by manual adjustment of the pressure-regulating tubing clamp. The separate circuit-closure clamp was used only to maintain the closed-loop configuration and prevent loss of perfusate. Outlet pressure (*P*_out_), recorded 1,000 mm upstream of the test device, was the pressure outcome reported in this study.” |

| **Reviewer comment** | 3. Where each device was positioned? Is this correct? This manuscript described that the distal tip of each device was positioned approximately 10 mm downstream of the roller pump, corresponding to the arterial segment of the mock CPB circuit. |
| --- | --- |
| **Response** | We thank the reviewer for pointing out this ambiguity. We rechecked the experimental setup and found that the previous wording could incorrectly suggest that the distal tip of the test device was positioned approximately 10 mm downstream of the roller pump. The 10-mm value actually referred to the insertion depth of the test device into the arterial-line tubing, not to its distance from the roller pump.  We therefore corrected the Materials and Methods section to state that each test device was inserted at the test-device site shown in Fig 1, with approximately 10 mm of its distal portion extending into the tubing lumen. We also added the same information to the Fig 1 caption. In addition, we clarified the definition of the length used for geometric comparison, distinguishing the measured 60-mm distal narrow-bore segment of the 8-Fr cannula from the effective lengths of the 14-G catheters and from the approximately 10-mm insertion depth used in the mock circuit. |
| **Manuscript changes** | We corrected the description of the test-device position in the Materials and Methods section and Fig 1 caption and clarified the distinction between device insertion depth and the lengths used for geometric comparison.  **Revised text in the Materials and Methods section, “Mock small-dog cardiopulmonary bypass circuit” subsection:**  “Each test device was inserted into the arterial-line tubing at the test-device site shown in Fig 1, such that approximately 10 mm of the distal portion of the device extended into the tubing lumen.”  **Clarified definition of the length used for geometric comparison:**  “The 60-mm length used for geometric comparison for the 8-Fr arterial cannula corresponded to the measured distal narrow-bore segment extending to the cannula tip and did not represent the overall device length. In contrast, for the 14-G catheters, effective length was defined as the axial distance from the catheter inlet to the distal tip/side-hole region. The selected catheter effective lengths were intended to represent device-length configurations relevant to toy-breed and small-dog applications.”  **Added text to the Fig 1 caption:**  “Each test device was inserted into the arterial-line tubing such that approximately 10 mm of its distal portion extended into the tubing lumen.” |

| **Reviewer comment** | 4. The conclusion section should be streamlined to better summarize the study's contributions and offer clear suggestions for future research. |
| --- | --- |
| **Response** | We thank the reviewer for this helpful suggestion. We agree that the Conclusions could be more concise and should more clearly distinguish the principal contribution of the study from the directions for future research. We therefore streamlined the Conclusions to focus on the circuit-level hydraulic findings that directly address the study objective, while retaining the important limitation that the present technical-replicate bench data do not establish device-level equivalence, interchangeability, or clinical validity. We also added a clear future-research statement emphasizing the need for multiple independent physical specimens, clinically relevant validation models, and formal equivalence testing when predefined device-level similarity is the intended conclusion. |
| **Manuscript changes** | We revised the Conclusions to provide a more concise summary of the principal circuit-level findings and to state the priorities for future research more explicitly.  **Revised Conclusions:**  “This in vitro bench study provides circuit-level hydraulic reference data for one 8-Fr pediatric arterial cannula and six 14-G hemodialysis catheter specimens at effective lengths of 25, 32, and 38 mm, with separate side-hole and sealed specimens at each length. Across the prespecified 50–300 mL/min pump flow-rate setting range, outlet-pressure differences between the three side-hole 14-G specimens and the 8-Fr cannula were small under the present bench conditions; at the 300 mL/min pump flow-rate setting, mean differences versus the 8-Fr cannula were +0.33 to +0.67 mmHg. Among the tested 14-G specimens, outlet pressure was 2.7–2.8 mmHg lower for the side-hole specimens than for the sealed specimens at 300 mL/min. At the exploratory 350 mL/min pump flow-rate setting, all three side-hole 14-G specimens showed higher outlet pressures than the 8-Fr cannula. These technical-replicate findings do not establish device-level equivalence, interchangeability, or clinical validity. Future studies should evaluate multiple independent physical specimens and clinically relevant validation models, with formal equivalence testing where predefined device-level similarity is the intended conclusion.” |

# Additional editorial corrections

In addition to the revisions made in direct response to the Academic Editor and reviewers, we corrected a mathematical notation error in the Table 4 note so that the Hagen–Poiseuille diameter scaling is correctly expressed as resistance ∝ *D*_i_^-4^. We also clarified that the S1 File contains trial-level *P*_out_ observations and updated the corresponding Zenodo DOI to 10.5281/zenodo.22293399. We also corrected the Fig 3 caption to describe the vertical reference line at 300 mL/min as dotted rather than dashed, consistent with the figure. We also refined the corresponding wording in the Discussion to describe the approximately 2.4 value as an “expected relative diameter-based resistance factor,” rather than as *D*_i_^-4^ alone, to clarify that this value is expressed relative to the 8-Fr cannula. We also corrected the numerical representation of the pressure-conversion factor in Equation 3 to 7.5006 × 10^-4^ so that it matches the factor stated in the accompanying text. We also shortened the Abstract to comply with the 300-word limit, revised the 300-mL/min pressure summary to approximately 136–137 mmHg, shortened the Fig 2 title to meet PLOS figure-caption guidance, and adjusted Fig 1 and Table 1 placement to PLOS manuscript-formatting requirements. We also corrected the Statistical analysis section by removing a statement regarding Shapiro–Wilk and Levene tests so that the text matches the reproducible analysis workflow provided in the S2 File. We also corrected minor bibliographic details in the reference list. Specifically, the authors’ initials in References 2 and 22 were corrected, the bibliographic information for Reference 18 was revised to match the original publication, and the DOI was added to Reference 23. These bibliographic and editorial corrections do not affect the numerical results, analyses, or interpretation of the study.
