# Supplementary material for: Hydraulic performance of 14-G hemodialysis catheters versus an 8-Fr arterial cannula in a mock 3-kg small-dog cardiopulmonary bypass circuit
Source: PLoS One. 2026 Oct 26;21(10):e0361473. doi: 10.1371/journal.pone.0361473 (PMC13704862; doi:10.1371/journal.pone.0361473)
Supplement: Supplementary file 6 [file pone.0361473.s006.docx]

**Comments to the Authors**

This is a clearly written in vitro bench study addressing a practical problem in cardiopulmonary bypass for toy-breed and small dogs. The experimental conditions are described in considerable detail, and I appreciate that the authors explicitly acknowledge several important limitations, including the use of technical rather than independent device-level replicates and the lack of evidence for formal equivalence or clinical interchangeability.

My comments are primarily from a clinical cardiovascular surgical and statistical perspective rather than from a detailed fluid-mechanical perspective. I believe the study may provide useful bench-reference data, but several issues regarding the clinical rationale and interpretation of the statistical analyses should be addressed.

**Major Revision**

1. **Clinical rationale for selection of the 8-Fr cannula as the comparator.** The clinical motivation for using a 14-G hemodialysis catheter is the limited arterial caliber in toy-breed dogs. However, the outer diameter of the 14-G catheter is 2.1 mm, which is substantially closer to that of the 6-Fr cannula (2.0 mm) than to that of the 8-Fr cannula (2.7 mm). The 6-Fr cannula is listed in Table 1 only as a reference and was not experimentally evaluated. Please clarify why the 8-Fr cannula was selected as the primary comparator and why the 6-Fr cannula was not tested. If the reason is that a 6-Fr cannula is expected to produce excessive resistance or cannot provide the required flow, this clinical rationale should be stated explicitly. I do not necessarily consider additional testing of the 6-Fr cannula mandatory, but the choice of comparator requires clearer justification.
2. **Interpretation of statistical inference from technical replicates.** The manuscript appropriately states that the six observations per condition are technical replicates obtained using the same physical device and circuit setup rather than six independent devices. Nevertheless, ANOVA, Welch's t-tests, confidence intervals, and standardized effect sizes are used extensively. These analyses primarily characterize repeatability within the particular bench setup and do not provide device-level population inference regarding 14-G catheters or 8-Fr cannulas in general. I suggest reconsidering the emphasis placed on inferential hypothesis testing and more clearly emphasizing descriptive estimates and measurement variability. If the intent is to make inference at the device-type level, multiple independent physical specimens of each device would be required.
3. **Absence of statistical significance should not be interpreted as evidence of equivalence or similarity.** The manuscript appropriately states that formal equivalence was not established. However, several passages emphasize the absence of a statistically significant device effect and the fact that the 95% confidence intervals span zero. Given the technical-replicate design, and more generally because a non-significant result does not establish equivalence, I recommend that the authors place greater emphasis on the observed absolute pressure differences and their uncertainty rather than on non-significant p values. Statements such as “no device effect” should be carefully limited to the present experimental setup.
4. **Statistical analysis of the side-hole experiment.** The side-hole and sealed conditions appear to have been evaluated using the same physical catheter, whereas the analysis uses Welch's two-sample t-test. Please clarify whether measurements under the two configurations were paired or otherwise blocked by experimental run. If a natural pairing exists, a paired or repeated-measures analysis may be more appropriate. More importantly, because these remain technical measurements on the same physical catheter, the statistical inference should again be limited to bench repeatability. I would also recommend emphasizing the absolute difference of approximately 2–3 mmHg (approximately 2%) rather than the very large Hedges' g values, which appear to be driven largely by the very small within-condition variability of the bench experiment.
5. **Clinical interpretation of the 300 mL/min reference flow and the term “supraphysiologic.”** The manuscript derives the 300 mL/min reference flow from a perfusion index of 1.4 L/min/m². Please provide slightly more clinical context for this choice, including how representative this flow is of current CPB practice in approximately 3-kg dogs under the stated hypothermic conditions. Unless there is strong clinical evidence that 350 mL/min is physiologically excessive in this setting, “above the prespecified flow range” or similar wording may be preferable to the broader term “supraphysiologic.”
6. **Comparison with hemodialysis pressure values should be reconsidered.** The Discussion compares the approximately 135-mmHg pressure observed in the mock CPB circuit with pressure values reported during hemodialysis vascular-access surveillance. The circuit configuration, pressure measurement location, access conditions, and clinical significance of these measurements differ considerably between hemodialysis and CPB. I am concerned that this numerical comparison could unintentionally imply that the observed pressure is clinically acceptable or safe. I suggest removing this comparison or substantially de-emphasizing it unless a stronger physiological rationale can be provided.

**Minor Revision**

7. **Inconsistent p values for the side-hole comparisons.** The Holm-adjusted p values reported in the Results text are 0.005, 0.003, and <0.001, whereas Table 3 reports 0.010, 0.006, and <0.001. Please verify the analysis and correct the inconsistent values.
8. **Figure 1 would benefit from a clear flow-direction arrow and clearer labeling of the pressure measurement sites.** From a clinical reader's perspective, it would be helpful to indicate explicitly which pressure corresponds to the regulated 60-mmHg site, which represents the reported outlet pressure, and the direction of perfusate flow. This would make the experimental setup considerably easier to understand.
9. **The statement that “some centers adopt 14-G hemodialysis catheters” may be somewhat stronger than the cited evidence supports.** The manuscript appears to cite a limited clinical report of this off-label strategy. Wording such as “off-label use has been reported” may more accurately reflect the available evidence unless broader clinical use can be documented.
10. **The physiological justification for the chosen blood-analogue viscosity could be refined.** The working-fluid viscosity was appropriately measured at 28°C and showed good repeatability. However, Reference 14 appears primarily to support the relationship between hemodilution and whole-blood viscosity rather than directly representing hypothermic, hemodiluted canine blood under CPB conditions. A more directly relevant reference would strengthen the physiological justification, or the authors could clarify that 2.8 cP was selected as an approximate rather than species- and temperature-specific blood analogue.
11. **Editorial correction.** In the first paragraph of the Limitations section, “Therefore” is repeated in consecutive sentences. Please revise for readability.

Overall, I consider the study potentially useful as a standardized bench comparison, particularly because the authors appropriately avoid claiming formal equivalence or clinical interchangeability. However, the statistical results should be reframed more clearly as measurements of repeatability within the tested physical devices and circuit rather than as population-level evidence regarding device types. Clarification of the clinical rationale for the comparator and flow conditions would also improve the manuscript substantially.
