# Supplementary material for: Hydraulic performance of 14-G hemodialysis catheters versus an 8-Fr arterial cannula in a mock 3-kg small-dog cardiopulmonary bypass circuit
Source: PLoS One. 2026 Oct 26;21(10):e0361473. doi: 10.1371/journal.pone.0361473 (PMC13704862; doi:10.1371/journal.pone.0361473)
Supplement: Peer review history

# Peer review: Decision Letter 0

**DOI:** 10.1371/journal.pone.0361473.r001

**Authors:** Muhammad Shakaib

2 Sep 2026

Dear Dr. Wauchi,

Thank you for submitting your manuscript to PLOS One. After careful consideration, we feel that it has merit but does not fully meet PLOS One’s publication criteria as it currently stands. Therefore, we invite you to submit a revised version of the manuscript that addresses the points raised during the review process.

Please submit your revised manuscript by Oct 17 2026 11:59PM. If you will need more time than this to complete your revisions, please reply to this message or contact the journal office at plosone@plos.org. When you're ready to submit your revision, log on to https://www.editorialmanager.com/pone/ and select the 'Submissions Needing Revision' folder to locate your manuscript file.

- A letter that responds to each point raised by the academic editor and reviewer(s). You should upload this letter as a separate file labeled 'Response to Reviewers'.
- A marked-up copy of your manuscript that highlights changes made to the original version. You should upload this as a separate file labeled 'Revised Manuscript with Track Changes'.
- An unmarked version of your revised paper without tracked changes. You should upload this as a separate file labeled 'Manuscript'.

If you would like to make changes to your financial disclosure, please include your updated statement in your cover letter. Guidelines for resubmitting your figure files are available below the reviewer comments at the end of this letter.

If applicable, we recommend that you deposit your laboratory protocols in protocols.io to enhance the reproducibility of your results. Protocols.io assigns your protocol its own identifier (DOI) so that it can be cited independently in the future. For instructions see: https://journals.plos.org/plosone/s/submission-guidelines#loc-laboratory-protocols. Additionally, PLOS One offers an option for publishing peer-reviewed Lab Protocol articles, which describe protocols hosted on protocols.io. Read more information on sharing protocols at https://plos.org/protocols?utm_medium=editorial-email&utm_source=authorletters&utm_campaign=protocols.

As the corresponding author, your ORCID iD is verified in the submission system and will appear in the published article. PLOS supports the use of ORCID, and we encourage all coauthors to register for an ORCID iD and use it as well. Please encourage your coauthors to verify their ORCID iD within the submission system before final acceptance, as unverified ORCID iDs will not appear in the published article. *Only*  the individual author can complete the verification step; PLOS staff *cannot*  verify ORCID iDs on behalf of authors.

We look forward to receiving your revised manuscript.

Kind regards,

Muhammad Shakaib, PhD

Academic Editor

PLOS One

**Journal Requirements:**

When submitting your revision, we need you to address these additional requirements.

1. Please ensure that your manuscript meets PLOS ONE's style requirements, including those for file naming. The PLOS ONE style templates can be found at

https://journals.plos.org/plosone/s/file?id=wjVg/PLOSOne_formatting_sample_main_body.pdf and

https://journals.plos.org/plosone/s/file?id=ba62/PLOSOne_formatting_sample_title_authors_affiliations.pdf

2. If the reviewer comments include a recommendation to cite specific previously published works, please review and evaluate these publications to determine whether they are relevant and should be cited. There is no requirement to cite these works unless the editor has indicated otherwise.

[Note: HTML markup is below. Please do not edit.]

Reviewers' comments:

Reviewer's Responses to Questions

**Comments to the Author**

1. Is the manuscript technically sound, and do the data support the conclusions?

Reviewer #1: Partly

Reviewer #2: Yes

**********

2. Has the statistical analysis been performed appropriately and rigorously?

Reviewer #1: No

Reviewer #2: Yes

**********

3. Have the authors made all data underlying the findings in their manuscript fully available?

The PLOS Data policy

Reviewer #1: Yes

Reviewer #2: Yes

**********

4. Is the manuscript presented in an intelligible fashion and written in standard English?

Reviewer #1: Yes

Reviewer #2: Yes

**********

Reviewer #1: Comments to the Authors

This is a clearly written in vitro bench study addressing a practical problem in cardiopulmonary bypass for toy-breed and small dogs. The experimental conditions are described in considerable detail, and I appreciate that the authors explicitly acknowledge several important limitations, including the use of technical rather than independent device-level replicates and the lack of evidence for formal equivalence or clinical interchangeability.

My comments are primarily from a clinical cardiovascular surgical and statistical perspective rather than from a detailed fluid-mechanical perspective. I believe the study may provide useful bench-reference data, but several issues regarding the clinical rationale and interpretation of the statistical analyses should be addressed.

Major Revision

1. Clinical rationale for selection of the 8-Fr cannula as the comparator. The clinical motivation for using a 14-G hemodialysis catheter is the limited arterial caliber in toy-breed dogs. However, the outer diameter of the 14-G catheter is 2.1 mm, which is substantially closer to that of the 6-Fr cannula (2.0 mm) than to that of the 8-Fr cannula (2.7 mm). The 6-Fr cannula is listed in Table 1 only as a reference and was not experimentally evaluated. Please clarify why the 8-Fr cannula was selected as the primary comparator and why the 6-Fr cannula was not tested. If the reason is that a 6-Fr cannula is expected to produce excessive resistance or cannot provide the required flow, this clinical rationale should be stated explicitly. I do not necessarily consider additional testing of the 6-Fr cannula mandatory, but the choice of comparator requires clearer justification.

2. Interpretation of statistical inference from technical replicates. The manuscript appropriately states that the six observations per condition are technical replicates obtained using the same physical device and circuit setup rather than six independent devices. Nevertheless, ANOVA, Welch's t-tests, confidence intervals, and standardized effect sizes are used extensively. These analyses primarily characterize repeatability within the particular bench setup and do not provide device-level population inference regarding 14-G catheters or 8-Fr cannulas in general. I suggest reconsidering the emphasis placed on inferential hypothesis testing and more clearly emphasizing descriptive estimates and measurement variability. If the intent is to make inference at the device-type level, multiple independent physical specimens of each device would be required.

3. Absence of statistical significance should not be interpreted as evidence of equivalence or similarity. The manuscript appropriately states that formal equivalence was not established. However, several passages emphasize the absence of a statistically significant device effect and the fact that the 95% confidence intervals span zero. Given the technical-replicate design, and more generally because a non-significant result does not establish equivalence, I recommend that the authors place greater emphasis on the observed absolute pressure differences and their uncertainty rather than on non-significant p values. Statements such as “no device effect” should be carefully limited to the present experimental setup.

4. Statistical analysis of the side-hole experiment. The side-hole and sealed conditions appear to have been evaluated using the same physical catheter, whereas the analysis uses Welch's two-sample t-test. Please clarify whether measurements under the two configurations were paired or otherwise blocked by experimental run. If a natural pairing exists, a paired or repeated-measures analysis may be more appropriate. More importantly, because these remain technical measurements on the same physical catheter, the statistical inference should again be limited to bench repeatability. I would also recommend emphasizing the absolute difference of approximately 2–3 mmHg (approximately 2%) rather than the very large Hedges' g values, which appear to be driven largely by the very small within-condition variability of the bench experiment.

5. Clinical interpretation of the 300 mL/min reference flow and the term “supraphysiologic.” The manuscript derives the 300 mL/min reference flow from a perfusion index of 1.4 L/min/m². Please provide slightly more clinical context for this choice, including how representative this flow is of current CPB practice in approximately 3-kg dogs under the stated hypothermic conditions. Unless there is strong clinical evidence that 350 mL/min is physiologically excessive in this setting, “above the prespecified flow range” or similar wording may be preferable to the broader term “supraphysiologic.”

6. Comparison with hemodialysis pressure values should be reconsidered. The Discussion compares the approximately 135-mmHg pressure observed in the mock CPB circuit with pressure values reported during hemodialysis vascular-access surveillance. The circuit configuration, pressure measurement location, access conditions, and clinical significance of these measurements differ considerably between hemodialysis and CPB. I am concerned that this numerical comparison could unintentionally imply that the observed pressure is clinically acceptable or safe. I suggest removing this comparison or substantially de-emphasizing it unless a stronger physiological rationale can be provided.

Minor Revision

7. Inconsistent p values for the side-hole comparisons. The Holm-adjusted p values reported in the Results text are 0.005, 0.003, and <0.001, whereas Table 3 reports 0.010, 0.006, and <0.001. Please verify the analysis and correct the inconsistent values.

8. Figure 1 would benefit from a clear flow-direction arrow and clearer labeling of the pressure measurement sites. From a clinical reader's perspective, it would be helpful to indicate explicitly which pressure corresponds to the regulated 60-mmHg site, which represents the reported outlet pressure, and the direction of perfusate flow. This would make the experimental setup considerably easier to understand.

9. The statement that “some centers adopt 14-G hemodialysis catheters” may be somewhat stronger than the cited evidence supports. The manuscript appears to cite a limited clinical report of this off-label strategy. Wording such as “off-label use has been reported” may more accurately reflect the available evidence unless broader clinical use can be documented.

10. The physiological justification for the chosen blood-analogue viscosity could be refined. The working-fluid viscosity was appropriately measured at 28°C and showed good repeatability. However, Reference 14 appears primarily to support the relationship between hemodilution and whole-blood viscosity rather than directly representing hypothermic, hemodiluted canine blood under CPB conditions. A more directly relevant reference would strengthen the physiological justification, or the authors could clarify that 2.8 cP was selected as an approximate rather than species- and temperature-specific blood analogue.

11. Editorial correction. In the first paragraph of the Limitations section, “Therefore” is repeated in consecutive sentences. Please revise for readability.

Overall, I consider the study potentially useful as a standardized bench comparison, particularly because the authors appropriately avoid claiming formal equivalence or clinical interchangeability. However, the statistical results should be reframed more clearly as measurements of repeatability within the tested physical devices and circuit rather than as population-level evidence regarding device types. Clarification of the clinical rationale for the comparator and flow conditions would also improve the manuscript substantially.

Reviewer #2: Major Comments

1.What is the maximum blood flow rate that the arterial cannula and the hemodialysis catheters can be provided? It is best to indicate it in table 1.

2.What's the relationship of the clamp position and the inlet pressure? This manuscript described that the inlet pressure was regulated at 60 mmHg by partially occluding the arterial tubing with a clamp; the clamp position was manually adjusted while monitoring the pressure display to maintain the target inlet pressure before each recording. Please provide a detailed explanation of the Mock small-dog cardiopulmonary bypass circuit, and indicate the direction of fluid flow in Figure 1.

3.Where each device was positioned ? Is this correct? This manuscript described that the distal tip of each device was positioned approximately 10 mm downstream of the roller pump, corresponding to the arterial segment of the mock CPB circuit.

4.The conclusion section should be be streamlined to better summarize the study's contributions and offer clear suggestions for future research.

**********

what does this mean?). If published, this will include your full peer review and any attached files.

If you choose “no”, your identity will remain anonymous but your review may still be made public.

**Do you want your identity to be public for this peer review?**  For information about this choice, including consent withdrawal, please see our Privacy Policy

Reviewer #1: **Yes:**  Yuki Kondo

Reviewer #2: No

**********

[NOTE: If reviewer comments were submitted as an attachment file, they will be attached to this email and accessible via the submission site. Please log into your account, locate the manuscript record, and check for the action link "View Attachments". If this link does not appear, there are no attachment files.]

To ensure your figures meet our technical requirements, please review our figure guidelines: https://journals.plos.org/plosone/s/figures

You may also use PLOS’s free figure tool, NAAS, to help you prepare publication quality figures: https://journals.plos.org/plosone/s/figures#loc-tools-for-figure-preparation.

NAAS will assess whether your figures meet our technical requirements by comparing each figure against our figure specifications.

Attachment Submitted filename: Comments.docx

# Author comment: Author response to Decision Letter 1

**DOI:** 10.1371/journal.pone.0361473.r002

17 Sep 2026

We thank the Academic Editor and the reviewers for their careful and constructive comments. We have addressed all comments point by point and revised the manuscript accordingly. A detailed response to each editor and reviewer comment, including the corresponding manuscript changes, has been uploaded separately as the “Response to Reviewers” file. The revised manuscript and a marked-up version with changes highlighted have also been uploaded.

Attachment Submitted filename: Response to Reviewers.docx

# Peer review: Decision Letter 1

**DOI:** 10.1371/journal.pone.0361473.r003

**Authors:** Muhammad Shakaib

13 Oct 2026

Hydraulic performance of 14-G hemodialysis catheters versus an 8-Fr arterial cannula in a mock 3-kg small-dog cardiopulmonary bypass circuit

PONE-D-26-17814R1

Dear Dr. Wauchi,

We’re pleased to inform you that your manuscript has been judged scientifically suitable for publication and will be formally accepted for publication once it meets all outstanding technical requirements.

Within one week, you’ll receive an e-mail detailing the required amendments. When these have been addressed, you’ll receive a formal acceptance letter and your manuscript will be scheduled for publication.

An invoice will be generated when your article is formally accepted. Please note, if your institution has a publishing partnership with PLOS and your article meets the relevant criteria, all or part of your publication costs will be covered. Please make sure your user information is up-to-date by logging into Editorial Manager at Editorial Manager® and clicking the ‘Update My Information' link at the top of the page. For questions related to billing, please contact billing support.

If your institution or institutions have a press office, please notify them about your upcoming paper to help maximize its impact. If they’ll be preparing press materials, please inform our press team as soon as possible -- no later than 48 hours after receiving the formal acceptance. Your manuscript will remain under strict press embargo until 2 pm Eastern Time on the date of publication. For more information, please contact onepress@plos.org.

Kind regards,

Muhammad Shakaib, PhD

Academic Editor

PLOS One

Additional Editor Comments (optional):

Reviewers' comments:

Reviewer's Responses to Questions

**Comments to the Author**

Reviewer #1: All comments have been addressed

Reviewer #2: All comments have been addressed

**********

2. Is the manuscript technically sound, and do the data support the conclusions?

Reviewer #1: Yes

Reviewer #2: Yes

**********

3. Has the statistical analysis been performed appropriately and rigorously?

Reviewer #1: Yes

Reviewer #2: Yes

**********

4. Have the authors made all data underlying the findings in their manuscript fully available?

The PLOS Data policy

Reviewer #1: Yes

Reviewer #2: Yes

**********

5. Is the manuscript presented in an intelligible fashion and written in standard English?

Reviewer #1: Yes

Reviewer #2: Yes

**********

Reviewer #1: I thank the authors for their detailed point-by-point responses and careful revision of the manuscript. The concerns raised in my previous review have been satisfactorily addressed. I appreciate the authors’ efforts to respond to my comments and revise the manuscript accordingly. I have no further comments or requests for revision and recommend acceptance of the manuscript in its current form.

Reviewer #2: (No Response)

**********

what does this mean?). If published, this will include your full peer review and any attached files.

If you choose “no”, your identity will remain anonymous but your review may still be made public.

**Do you want your identity to be public for this peer review?**  For information about this choice, including consent withdrawal, please see our Privacy Policy

Reviewer #1: **Yes:**  Yuki Kondo

Reviewer #2: No

**********

# Editor report: Acceptance letter

**DOI:** 10.1371/journal.pone.0361473.r004

**Authors:** Muhammad Shakaib

PONE-D-26-17814R1

PLOS One

Dear Dr. Wauchi,

I'm pleased to inform you that your manuscript has been deemed suitable for publication in PLOS One. Congratulations! Your manuscript is now being handed over to our production team.

At this stage, our production department will prepare your paper for publication. This includes ensuring the following:

* All references, tables, and figures are properly cited

* All relevant supporting information is included in the manuscript submission,

* There are no issues that prevent the paper from being properly typeset

You will receive further instructions from the production team, including instructions on how to review your proof when it is ready. Please keep in mind that we are working through a large volume of accepted articles, so please give us a few days to review your paper and let you know the next and final steps.

Lastly, if your institution or institutions have a press office, please let them know about your upcoming paper now to help maximize its impact. If they'll be preparing press materials, please inform our press team within the next 48 hours. Your manuscript will remain under strict press embargo until 2 pm Eastern Time on the date of publication. For more information, please contact onepress@plos.org.

You will receive an invoice from PLOS for your publication fee after your manuscript has reached the completed accept phase. If you receive an email requesting payment before acceptance or for any other service, this may be a phishing scheme. Learn how to identify phishing emails and protect your accounts at https://explore.plos.org/phishing.

If we can help with anything else, please email us at customercare@plos.org.

Thank you for submitting your work to PLOS One and supporting open access.

Kind regards,

PLOS One Editorial Office Staff

on behalf of

Dr. Muhammad Shakaib

Academic Editor

PLOS One
